# Validation of remote multimodal AI screening for Parkinson disease across diverse settings

**DOI:** 10.1038/s43856-026-01606-6

**Published:** 2026-05-06

**Authors:** Md Saiful Islam, Tariq Adnan, Abdelrahman Abdelkader, Zipei Liu, Evelyn Ma, Sooyong Park, Asif Azad, Pai Liu, Meghan Pawlik, Emily Hartman, Erin Shelton, Kristina B. Larson, M. Saifur Rahman, Cathe Schwartz, Karen Jaffe, Jamie L. Adams, Ruth B. Schneider, Jan Freyberg, E. Ray Dorsey, Ehsan Hoque

**Affiliations:** 1 University of Rochester, Rochester, NY USA; 2 Bangladesh University of Engineering & Technology, Dhaka, Bangladesh; 3 Atria Health and Research Institute, New York, NY USA; 4 University of Rochester Medical Center, Rochester, NY USA; 5 InMotion, Beachwood, OH USA; 6 Harvard Medical School, Boston, MA USA; 7 Google Research, London, UK; 8 Ministry of Defense, Riyadh, Saudi Arabia

**Keywords:** Parkinson's disease, Parkinson's disease

## Abstract

**Background:**

Timely detection of Parkinson’s disease (PD) remains limited by reliance on in-person neurological evaluations that are often costly and geographically inaccessible. To address these barriers, we develop PARK (*Parkinson’s Analysis with Remote Kinetic-tasks*) – a web-based artificial intelligence (AI) tool that screens for PD using short webcam recordings of facial expression, motor, and speech tasks.

**Methods:**

Across eight independent studies (*n* = 1,865 participants; 670 with PD), participants completed three standardized tasks (smile mimicry, finger tapping, and pangram utterance) via webcam. Task-specific neural networks estimate PD risk and uncertainty, which are integrated through an uncertainty-calibrated fusion model (UFNet). Model performance is evaluated on one internal and two external test sets representing supervised and unsupervised real-world environments. Three movement disorder specialists also reviewed videos from 30 participants to benchmark clinical agreement of the PARK tool. User experience is assessed through structured surveys containing open-ended or multiple-choice questions.

**Results:**

PARK achieves accuracies of 80.2–80.6% and AUROC of 0.85-0.87 across all evaluation cohorts, with 83.3–86.5% sensitivity and 71.2–78.4% specificity. Predictive performance remains stable across sex, age, and ethnicity. Agreement with clinician judgments reaches Cohen’s *κ* = 0.59. Uncertainty estimates reflect diagnostic confidence, and performance declines at high-uncertainty levels. Usability is rated highly (System Usability Scale  > 70) in both supervised and unsupervised settings, with low perceived risk and strong user preference for remote screening.

**Conclusions:**

PARK demonstrates promising accuracy and favorable user acceptance for remote PD screening, highlighting its potential as an accessible, equitable, and uncertainty-aware tool for neurological assessment when traditional care is challenging to obtain.

## Plain Language Summary

Parkinson’s disease is a movement disorder that can be difficult to detect, especially for people who live far from clinics or have limited access to specialists. This study develops PARK, an easy-to-use online tool that uses a computer’s camera and microphone to analyze short videos of facial expressions, finger tapping, and speech. The tool uses artificial intelligence to identify signs of Parkinson’s disease and estimate how likely this is to indicate the presence of Parkinson’s disease. In tests with 1,865 people, PARK identifies individuals with Parkinson’s with over 80% accuracy and is well-liked by users. The findings suggest that tools such as PARK can help more people receive timely screening and guidance, especially in areas with limited access to neurological care.

## Abstract (web summary)

Islam, Adnan and Abdelkader develop PARK, an artificial intelligence tool that screens for Parkinson’s disease using short webcam recordings of facial, motor, and speech tasks. Across 8 studies involving 1,865 participants, there is 80% accuracy in detecting Parkinson’s disease and strong agreement with clinician assessments.

## Introduction

Parkinson’s disease (PD), one of the fastest-growing neurological disorders^1^, imposes a substantial and growing economic burden, with estimated costs in the United States exceeding $50 billion^2^. Yet timely detection remains out of reach for many^3,4^, as diagnosis typically relies on in-person neurological evaluations that are often costly, time-consuming, and inaccessible, particularly in underserved regions^5,6^. Even where care is available, long wait times and limited access to specialists delay diagnosis and intervention^7,8^. Because early identification may help preserve quality of life^9^, there is an urgent need for scalable, non-invasive screening tools that can reach individuals before symptoms become disabling.

Several approaches have emerged to facilitate PD assessment outside the clinic, including wireless and wearable sensors^10–13^, speech-based tools^14,15^, and video analysis^16–21^. While these methods show promise, they are often constrained by cost, complexity, or limited scope (e.g., relying on a single task or modality). Biomarker-based diagnostics, such as the cerebrospinal fluid *α*-synuclein seed amplification assay^22^, demonstrate high diagnostic accuracy but remain invasive and logistically challenging for broad deployment. Many studies also draw from small, demographically narrow cohorts, limiting generalizability. Given the heterogeneity of PD symptoms^23^, robust, accessible, and scalable multimodal tools are essential to capture the full spectrum of PD manifestations.

In prior work, we introduced UFNet^24^, a lightweight multimodal model that analyzes short webcam recordings of three standardized tasks (speech, facial expression, and finger tapping) to screen for PD. In this study, we advance UFNet from a promising algorithmic framework to a patient-centered screening tool named *Parkinson’s Analysis with Remote Kinetic-tasks* (PARK). Across eight studies including 1,865 participants, PARK demonstrates consistent screening performance across diverse demographic groups and real-world settings. The system achieves 80.2–80.6% accuracy, with strong sensitivity (83.3–86.5%) and specificity (71.2–78.4%), shows moderate agreement with specialist assessments (*κ* = 0.59), and maintains stable performance across age, sex, and ethnicity. Participants report high usability and acceptability of the system (average System Usability Scale^25^ score of 70.8–75.5) in both supervised and unsupervised environments. Together, these findings suggest that remote multimodal AI screening may provide a practical and accessible approach for identifying individuals who could benefit from further neurological evaluation.

## Methods

### Description of data sources

The data used in this work were compiled from eight independent studies described below. Each study received approval from the institutional review board (IRB) of the corresponding institution(s) (see Table 1 for details). Individuals whose images appear in the manuscript provided explicit e-consent for publication of identifiable images. Please see the “Data Availability” section for details regarding data access.

**Table 1:** IRB-approved study protocols

Studies	Approving Institution	IRB Number
Parktest, Parktest@InMotion	University of Rochester	RSRB#00001369
ROOSTER-PD	University of Rochester Medical Center	RSRB#00003703
SuperPD	University of Rochester Medical Center	RSRB#00001787
Cluster-PD	University of Rochester Medical Center	RSRB#00004849
ROUTE-PD	University of Rochester	RSRB#00008275
Validation Study-1,2	University of Rochester	RSRB#00006474

This table lists the studies whose data are used in this work, along with the approving institution and corresponding Institutional Review Board (IRB) protocol numbers. All studies were reviewed and approved by the University of Rochester or the University of Rochester Medical Center to ensure compliance with ethical guidelines for research involving human participants.

**1. Parktest (2017-Ongoing)**: Parktest is a non-clinical study aimed at collecting multimodal video and audio recordings from individuals with and without PD via a publicly accessible web-based platform (https://parktest.net)^26^. Participants were recruited globally through multiple channels, including the University of Rochester Brain Health Registry, clinician referrals, and social media outreach, ensuring broad geographic and demographic representation. Enrollees completed multiple standardized tasks, including the three analyzed in this study, designed to mimic components of the MDS-UPDRS^23^. All recordings were conducted at home without supervision, and instructional videos were provided before each task. Given the non-clinical nature of the study, PD diagnosis (PD or non-PD) was entirely self-reported and not clinically confirmed. Participants provided informed e-consent (collected via the web-based platform) for their data to be used in this study.

**2. SuperPD (2020–2022)**: SuperPD was a longitudinal clinical study investigating PD progression over a two-year period. Participants with and without PD were enrolled from the greater Rochester, New York area through the University of Rochester Medical Center (URMC). Participants completed standardized tasks at baseline, 6, 12, and 24 months using the same web-based platform. Therefore, each individual could contribute up to four data sessions across the study period. Data were collected under supervision, and all PD diagnoses were confirmed using the UK PD Society Brain Bank Diagnostic Criteria^27^. A study coordinator collected participants’ informed consent for their data to be used in this study.

**3. ROOSTER-PD (2021-Ongoing)**: ROOSTER-PD is a longitudinal sub-study embedded within a six-year remote observational study of individuals carrying the LRRK2 G2019S variant, which is the most common autosomal dominant cause of PD^28^. Participants in the parent study, VALOR-PD^29^, were recruited nationwide through 23andMe (https://23andme.com). Participants enrolled in the ROOSTER-PD sub-study complete annual remote assessments using the web platform, with study staff providing supervision as needed during video data collection. Participants included non-manifest carriers without a clinical PD diagnosis and manifest carriers with a clinical PD diagnosis. Participants provided informed consent to use their data in this study.

**4. Cluster-PD (2021–2023)**: Cluster-PD was a cross-sectional clinical study evaluating environmental exposure as a potential PD risk factor. The study enrolled attorneys from Monroe County (New York). A subset of the attorneys worked adjacent to a site with known trichloroethylene (TCE) and perchloroethylene (PCE) contamination, which are associated with an increased risk of PD^30,31^. Participants completed the standard PARK tasks under clinical supervision, and PD diagnoses were clinically verified using the Gelb Diagnostic Criteria^32^. We obtained informed consent from all participants for using their data in this study.

**5. Parktest@InMotion (2022–2023)**: For this study, we specifically reached out to the clients of InMotion (a PD wellness center in Ohio, United States) with a clinically confirmed PD diagnosis. We collected their informed consent and asked them to complete the standard PARK tasks. A control group of individuals without PD (clients’ caregivers) was also recruited. Participants completed the tasks onsite and were supervised by the staff at the InMotion facility as needed.

**6. ROUTE-PD (2023–2024)**: ROUTE-PD was a one-year longitudinal study conducted at InMotion, aimed at tracking symptom progression through monthly assessments. Participants with and without PD completed the standard PARK tasks, primarily without supervision. On-demand assistance from staff was available. All PD diagnoses were confirmed clinically at enrollment and re-evaluated at study conclusion using the MDS Clinical Diagnostic Criteria for PD^33^. Informed e-consent was obtained from all participants at enrollment for the use of their data in this study.

**7. Validation Study-1 (2022–2023)**: Validation Study-1 was conducted to evaluate the usability and real-world performance of the PARK tool in a supervised community setting. InMotion clients (with PD) and their caregivers (without any diagnosed movement disorder) who did not participate in any prior studies relevant to this work were invited to enroll, ensuring our model did not see any data from these participants during training and development. In addition to completing the standardized tasks, participants completed usability surveys (both qualitative and quantitative) and provided feedback on the potential risks and benefits of the PARK tool. All participants were supervised by the study staff, and participants with PD had their diagnoses confirmed by a movement disorders specialist. Participants provided informed e-consent (collected via the PARK tool’s web interface) for their data to be used in this study.

**8. Validation Study-2 (2024–2025)**: Validation Study-2 was a replication of Validation Study-1 conducted in an unsupervised, home-based setting. Participants with and without PD were recruited nationwide (within the United States) and completed the standardized tasks and follow-up surveys using the web platform. In addition to the survey questions used in Validation Study-1, we included Likert-scale^34^ questions on the potential risks and benefits of the PARK tool, to assess these aspects quantitatively. Inclusion criteria included the ability to provide informed consent and either a PD diagnosis (confirmed by a movement disorders specialist) or self-reported absence of any movement disorder. Prior participation in PARK studies was an exclusion criterion to ensure the model was tested on unseen individuals. All sessions were completed independently, without supervision.

### Data pre-processing

Many videos were recorded in unsupervised home environments, resulting in varying levels of quality and compliance. While not all quality issues could be automatically detected, we applied several exclusion criteria to ensure data integrity. Specifically, we automatically discarded videos with the following quality issues:

- videos with abnormal durations (defined as more than three standard deviations below or above the average duration of the respective task videos),
- videos in which the relevant body part was not visible (e.g., obscured hands during the finger-tapping task),
- videos unreadable by the FFmpeg library^35^, and
- videos from which task-relevant computational features could not be extracted.

Participants and their video data were removed if they did not contribute at least one task-video (not necessarily all three tasks) that satisfied these quality criteria. All remaining videos were standardized to 15 frames per second (fps), resized to 640 × 480 resolution, and encoded in MP4 format (H.264 codec).

### Dataset splits

**Fig. 1:**
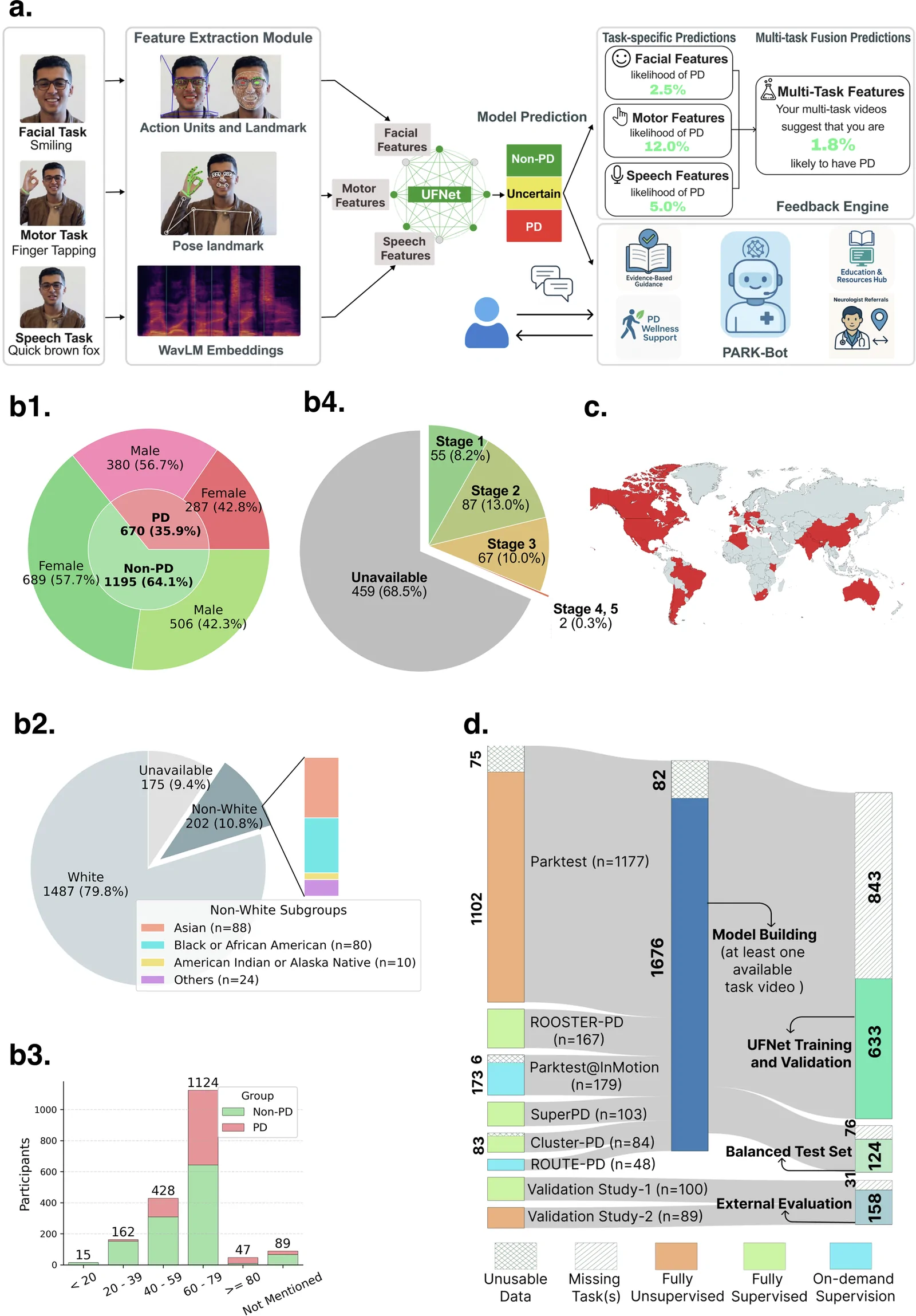
Study overview. **a** Overview of the PARK screening tool. Users complete three standardized tasks (facial expression, finger tapping, and speech) via a webcam. Landmark trajectories and speech features are extracted using MediaPipe^41^, OpenFace^44^, and WavLM^46^, and summarized into clinically relevant features. Task-specific neural networks estimate PD risk and uncertainty, which are integrated by the UFNet fusion model^24^ to generate an overall prediction. The system withholds uncertain outputs and provides task-level assessments, educational resources, and a large language model-powered chatbot for user feedback and support. **b** Demographic characteristics of study participants, including: **b1** joint distribution of sex and PD diagnosis; **b2** ethnicity distribution; **b3** age distribution; and **b4** distribution of Hoehn and Yahr Parkinson’s disease stages. **c** Geographic distribution of study participants. Countries highlighted in red represent locations from which participants contributed data, illustrating the global reach of the study cohort. **d** Dataset composition and study splits. Participants from six independent studies were used to construct the training, development, and balanced test sets, with strict separation of individuals across splits. Two additional studies, comprising entirely new participants, were reserved for external evaluation of the PD screening model. The color coding of the bars on the left indicates the level of supervision involved in data collection across the studies. Double-cross-out grids indicate cohorts that contributed no usable video data. Single cross-out grids indicate participants who provided at least one usable task video but did not complete all three tasks within a single session. Data from participants of the latter group were still used to train task-specific models. Only participants who completed all three task videos in at least one session were used for UFNet training and evaluation. The authors have obtained consent to publish the images of the human subject.

We used data from the first six studies (Parktest, SuperPD, ROOSTER-PD, Cluster-PD, Parktest@InMotion, and ROUTE-PD) for model training, selection, and evaluation (see Fig. 1d for details). From this pool, we randomly sampled 120 participants for model selection, and another 200 for testing, ensuring class balance (PD vs. non-PD) and broad demographic coverage. The remaining participants were assigned to the training set.

To ensure balanced demographic representation, we used a genetic algorithm^36^ to maximize diversity within each sampled cohort (except for the training set). We initialized the algorithm with a random set of 50% PD and 50% non-PD participants. We enforced a random, artificial mutation at each iteration–replace a random participant within the set with another random participant (with the same PD/non-PD classification) outside the set. The mutation was accepted if it increased the demographic diversity of the participants. Specifically, for each set, we defined expected distributions across sex (50% male, 50% female), ethnicity (45% white, 45% non-white, and 10% unknown), and age (45% below 50 years, 45% above 50 years, and 10% unknown) subgroups. We then proposed a diversity loss ${{{\mathcal{L}}}}_{diversity}$ that penalized when the observed counts in each subgroup differed from the expected counts.

1 $${{{\mathcal{L}}}}_{diversity}={\sum }_{a\in C}{\sum }_{i\in a}\frac{({E}_{i}-{O}_{i})^{2}}{{E}_{i}}$$

Here, *C* denotes different demographic attributes (e.g., sex, ethnicity, and age), *i* denotes the subgroups of an attribute *a* (e.g., male, female), and *E*_*i*_ and *O*_*i*_ denote the expected and observed numbers of participants, respectively, for the target subgroup. Smaller differences are less penalized, while the larger differences are heavily penalized due to the quadratic nature of ${{{\mathcal{L}}}}_{diversity}$. Reduced loss corresponds to increased demographic diversity. Eventually, the genetic algorithm tries to minimize this loss and converges to reach a local optimum. To approximate the global optimum, the algorithm was run 500 times, and the solution with the minimum diversity loss was chosen. First, 200 participants (100 with PD vs. 100 without; 49% male vs. 51% female; 45% white vs. 24% Non-white and 31% unknown; 29% below 50 years vs. 59% above 50 years and 12% unknown) were chosen for model testing (termed as Balanced Test Data throughout the article) out of the entire population of six studies. Then, the 200 participants selected were excluded, and the process was re-run to select the 120 participants for model selection.

Participants from two separate validation studies (Validation Study-1 and Validation Study-2) were reserved for real-world evaluation of the final (locked) model. These are referred to as External Evaluation (Supervised) and External Evaluation (Unsupervised), respectively, throughout this paper.

### Task selection

Parkinsonian symptoms can be multifaceted and often manifest across multiple modalities—facial expressions, motor functions, and speech. To analyze multiple modalities simultaneously, we collected audio and video recordings of three standardized tasks: finger tapping (motor function), smile-mimicry (facial expressions), and pangram (i.e., a sentence containing all the letters of the alphabet) utterance (speech). The finger-tapping task requires participants to tap their thumb with their index finger 10 times, performing the motion as fast and large as possible. In the smile-mimicry task, participants are instructed to smile naturally in front of the camera, then gradually return to a neutral facial expression, repeating this sequence three times. In the pangram utterance task, participants are asked to read aloud a sentence containing all 26 letters of the English alphabet. For this study, we used the widely-used^14,15^ pangram: “The quick brown fox jumps over the lazy dog.”

The selection of these tasks was guided by considerations of clinical relevance, feasibility for at-home deployment, and participant safety. Although gait analysis is a well-established method for assessing PD severity and progression^11,37^, its remote implementation poses significant challenges. Accurately capturing gait requires full-body visibility, adequate walking space, and controlled environments, all of which are difficult to guarantee in unsupervised home settings. Moreover, the physical exertion involved may increase the risk of falls among individuals with advanced motor symptoms. Similarly, sustained phonation–where participants are asked to hold a vowel sound for as long as possible–has shown promise in PD detection^38,39^. However, its utility is often hindered by variability in microphone sensitivity and ambient noise, complicating downstream acoustic analysis. In contrast, the selected tasks can be performed while seated, require minimal equipment, and are robust to environmental variations, making them well-suited for scalable, at-home screening^24^.

### Extracting computational features

Deep learning models have shown remarkable success in learning representations from raw, non-tabular inputs such as video and audio. However, their performance typically depends on access to large-scale, annotated datasets; for example, state-of-the-art cancer detection systems are often trained on hundreds of thousands of samples^40^. Given the limited size of our dataset of patient-recorded videos, we adopted a feature engineering approach informed by prior literature and domain expertise. Rather than relying solely on data-intensive deep learning models, we extracted clinically meaningful, task-specific features tailored to each standardized neurological task: finger tapping, facial expression, and speech. These features have been validated in previous studies and are robust to the recording variability observed across both home and clinical settings. While we refer the reader to previous work for detailed definitions and validation of these features, we briefly summarize them below.

*Finger-tapping Task*: Inspired by Islam et al.^18^, we utilized MediaPipe^41^ to extract 65 features per hand to objectively analyze bradykinesia^42^ and rigidity through features such as tapping speed, amplitude, and interruptions. This extraction process yielded 130 features for the finger-tapping task to model motor impairments.

*Smile Task*: Adnan et al.^43^ identified 42 facial features for analyzing smile mimicry videos. These features, extracted using OpenFace^44^ and MediaPipe, captured digital markers for PD closely aligned with the MDS-UPDRS^23^ guidelines for in-person evaluation. Examples include intensity of facial muscle movements, smile spontaneity, eye blinking, lip separation, etc. Machine learning models trained solely on this feature set demonstrated promising performance, indicating the effectiveness of these features in detecting PD-related changes in facial expressions (such as hypomimia^45^).

*Speech (Pangram Utterance) Task*: For the speech task, we extracted 1024-dimensional embeddings using WavLM^46^, a self-supervised speech representation model pre-trained on a large-scale, diverse audio corpus. These embeddings capture fine-grained acoustic features, including prosody, articulation, and vocal quality, that are frequently impaired in individuals with PD. In our prior work^15^, we demonstrated that WavLM-based embeddings significantly outperformed conventional handcrafted features such as pitch, jitter, and shimmer, highlighting their utility for PD screening in real-world, unconstrained environments.

### Model training

We followed the training protocol established in our prior work^24^, which involves a two-stage pipeline: task-specific modeling followed by multimodal fusion using the Uncertainty-calibrated Fusion Network (UFNet). Task-specific models were trained on all available data from the training-set participants. For individuals who contributed multiple sessions over time, particularly in longitudinal studies such as ROOSTER-PD and ROUTE-PD, each session was labeled according to the most recent PD diagnosis status as of the session date. UFNet was trained exclusively on sessions where all three task videos (motor, facial, and speech) were present and met quality criteria.

In the first stage, each of the three tasks–finger-tapping, smile, and pangram utterance–is independently modeled using shallow neural networks tailored to the domain-specific features. All task-specific models were trained with binary cross-entropy loss, employed ReLU activations (with sigmoid on the output), and used either SGD or AdamW as the optimizer. Importantly, dropout was applied during both training and inference to quantify uncertainty by aggregating predictions over multiple stochastic forward passes.

In the second stage, the UFNet model integrates the outputs from the three task-specific networks in an uncertainty-aware fashion. Each task’s features are first projected to a shared dimensional space using linear transformations, followed by ReLU activation, Monte Carlo (MC) dropout, and layer normalization. These projected vectors are then combined using a calibrated self-attention mechanism, in which attention weights are modulated by task-specific uncertainties to downweight unreliable modalities. The contextualized representations from self-attention, along with the mean prediction probabilities from the task-specific models, are concatenated and passed through another shallow neural network trained with MC dropout. To ensure safe deployment in clinical screening settings, UFNet incorporates a mechanism to withhold predictions for uncertain cases. Specifically, predictions are flagged as uncertain if the 95% confidence interval from MC dropout includes both PD and non-PD classes, preventing potentially misleading outputs.

This two-stage architecture enables the model to robustly integrate heterogeneous signals from motor, facial expression, and speech modalities while accounting for task-specific reliability. Also, UFNet has only 226,000 trainable parameters, making it suitable for training on smaller datasets and deployment on lightweight devices such as personal computers.

### Model selection and performance reporting

Both the task-specific models and the UFNet employ shallow neural network architectures and require tuning of several hyperparameters, including learning rate, batch size, dropout probability, and optimizer settings. Given the class imbalance in the training set (more non-PD than PD samples), we also explored the utility of minority oversampling techniques such as SMOTE^47^. Task-specific features were further processed through multiple configurations of feature scaling (e.g., standardization, min-max normalization) and feature selection (e.g., correlation-based filtering). We employed Weights & Biases (https://wandb.ai) to conduct an efficient hyperparameter search (see Supplementary Information for the complete search space), using Bayesian optimization to maximize the Area Under the Receiver Operating Characteristic (AUROC) on the model selection dataset.

Due to the two-stage architecture, we first performed hyperparameter tuning independently for each task-specific model, selected the best-performing configuration on the model selection set, and then locked those models. The outputs from these locked task-specific models were then used as inputs to UFNet, for which a separate round of hyperparameter tuning was conducted. The final UFNet model, selected based on the highest AUROC on the model selection set, was evaluated on the balanced test data and the two external validation sets (supervised and unsupervised) independently.

Model performance was assessed using metrics critical for clinical evaluation: AUROC, Area Under the Precision-Recall Curve (AUPRC), Accuracy, *F*_1_-score, Sensitivity, Specificity, Positive Predictive Value (PPV), and Negative Predictive Value (NPV). To assess the reliability of model-predicted probabilities, we also evaluated calibration using Expected Calibration Error (ECE)^48^ and Brier Score^49^, ensuring that model predictions align with the true likelihood of Parkinsonian disorder.

### Specialist assessments

To benchmark the PARK tool’s performance against clinical expertise, we conducted a comparative evaluation using a curated set of 30 participants randomly drawn from the validation cohorts (Balanced Test Data, External Evaluation (supervised), and External Evaluation (unsupervised)). All participants had completed the full set of standard PARK tasks (finger-tapping, smile, and speech).

Three experienced movement disorder specialists, each an associate or full professor in the Department of Neurology at the University of Rochester Medical Center, with over a decade of clinical experience diagnosing and managing Parkinsonian disorders, independently reviewed these recordings. However, full in-person clinical assessments were not feasible for this cohort due to logistical and ethical considerations. Participants were geographically dispersed and recruited remotely, making it difficult to trace and invite them for follow-up assessments. Moreover, re-contacting participants solely for clinical evaluation was not covered under the original IRB protocols. As a result, the specialists based their evaluations exclusively on the video recordings of the three standardized tasks, without access to clinical history or neurological exams. This constraint mirrors the real-world deployment setting of the PARK tool, which also operates solely on task recordings.

Each specialist independently assessed whether the participant exhibited symptoms consistent with a Parkinsonian disorder. The same recordings were simultaneously processed by the PARK model, which had been locked prior to evaluation. Performance was quantified using standard diagnostic metrics: accuracy, sensitivity, specificity, positive predictive value (PPV), negative predictive value (NPV), and *F*_1_ score. To contextualize PARK’s performance relative to clinical judgment, we also computed inter-rater agreement among the specialists and between each specialist and the PARK tool using Cohen’s kappa.

### Development of PARK chatbot

To complement the AI-based remote screening provided by the PARK tool, we developed an LLM-driven chatbot (PARK chatbot) to support participants immediately after the presentation of their screening results. The chatbot was built on a GPT-3.5 backend and integrated into the web platform used in both Validation Study-1 and Validation Study-2. The chatbot served as an interactive guide to help users interpret the screening outcomes, access curated educational materials, and identify local resources for follow-up care. Specifically, it was configured to explain screening results in plain language and to direct users to trusted resources, including the Movement Disorder Society (MDS) directory of neurologists and local support groups.

To avoid potential distress, we added guardrails to the chatbot to ensure it provides consistent, appropriate responses and does not provide clinical advice (e.g., medication). To achieve this, the system was constrained through a set of strict behavioral instructions that enforced the following:

- No responses to questions about medications or treatment plans
- No interpretation of symptoms as a medical diagnosis
- No provision of clinic-specific or time-sensitive information
- No engagement in off-topic dialogue

In these cases, the chatbot provided links to verified external sources such as the Parkinson’s Foundation. In addition, the chatbot is designed to reiterate that the PARK tool is a screening tool, not a diagnostic platform, and that all health decisions should be made in consultation with a qualified clinician. The guardrails were enforced through a structured prompt.

### User-centric assessments

To evaluate the usability, perceived benefits and risks, and user preferences associated with the PARK tool when used in home or community environments, we conducted two independent user studies-Validation Study-1 and Validation Study-2. In addition to capturing video recordings of participants performing standard PARK tasks, we administered structured surveys to quantify user feedback across multiple dimensions. The surveys were designed after discussions with multiple stakeholders involved in PD care: three neurologists, individuals with PD and their caregivers (without PD), and a bioethics specialist.

Validation Study-1 was conducted in a supervised community setting where participants interacted with the PARK tool while receiving assistance from onsite staffs familiar with the task requirements and technology. In contrast, Validation Study-2 was designed to more closely reflect real-world conditions, in which participants completed the protocol independently in their home environments without direct supervision.

Note that, due to protocol variances and differing timelines in these two studies, quantitative responses to some of the sections (marked with a *) were only collected in Validation Study-2, while Validation Study-1 asked open-ended questions to assess these categories. Although responses to the open-ended questions provided insights, they were not included in the quantitative analyses discussed in this paper. The detailed list of survey questionnaires is provided in the Supplementary Information.

- *Usability*: All participants completed a series of Likert-scale survey items assessing usability and system functionality, including a modified System Usability Scale (SUS). Questions addressed the ease of task completion, the clarity of instructional materials, and the intuitiveness of interacting with the PARK chatbot (e.g., “The instructional videos helped me to complete the tasks; The tasks were easy to complete; It was easy to interact with PARK-Bot”).
- *Utility and Comprehensiveness of Resources*: To evaluate the utility and comprehensiveness of educational and interpretive resources provided by the tool, participants responded to questions concerning the clarity and informativeness of risk descriptions and PD likelihood, as well as the adequacy of answers provided by PARK-Bot.
- Preference Compared to Clinical Appointments*: Participants compared their experience using PARK to traditional in-person clinical assessments in terms of accessibility, convenience, comfort, privacy, and financial impact.
- Perceived Risks and Benefits*: Questions in this section captured emotional responses to the PARK results, including feelings of anxiety or empowerment, and concerns regarding the potential harm of automated responses.
- Preference for Using the PARK chatbot*: Participants indicated their preferences for interacting with the conversational agent across different contexts (e.g., for ease-of-use, comfort, or safety).

Survey responses provided insight into the real-world usability and perceived value of PARK across different supervision conditions, helping to identify barriers and facilitators to adoption in diverse settings.

### Video quality analysis

To ensure data integrity and the reliability of downstream analyses, we conducted a comprehensive quality assessment of all video recordings, including audio, smile, and finger-tapping tasks. Three human annotators (two doctoral students and one undergraduate student with 2+ years of experience in PD video analysis) performed the evaluations. All annotators had 2+ years of experience developing and interacting with the PARK framework, were knowledgeable about clinical PD assessments and MDS-UPDRS criteria, and were well-acquainted with the dataset, thereby ensuring credibility and consistency.

In the initial phase, annotators independently assessed 50 randomly selected recordings for each task and rated video quality on a three-point scale: 0 (poor quality), 1 (ambiguous), and 2 (high quality). They then met to resolve discrepancies and establish a standardized guideline to maintain labeling consistency. This process facilitated a set of clearly defined criteria for the annotations (see Supplementary Information for detailed criteria). In summary, for audio recordings, the annotators evaluated background and microphone noise, unnatural pauses, and verbal compliance, without incorporating visual features. In contrast, smile and finger-tapping videos were assessed based on visibility, background contrast, participant positioning, and adherence to task instructions. Final quality annotations of the videos used for evaluating PARK were based on the collaboratively developed guidelines. The “ambiguous” category was applied sparingly and only in cases with conflicting quality indicators.

### Explainability analysis with SHAP

We computed post hoc explanations on the final, frozen UFNet model using SHAP^50^. Explanations were evaluated on the combined test set (number of participants, *n* = 320) with a background distribution summarized from the *training* features using k-means. The model receives, for each modality (finger, speech, smile), a feature block along with that modality’s unimodal prediction and predictive uncertainty (Monte Carlo dropout mean and variance). To interface with SHAP, we used a wrapper that reconstructs the model’s tuple input from a flat design matrix (features ∣ predictions ∣ uncertainties) and disabled dropout at inference.

We used Kernel SHAP with a logit link, a k-means background, and a sampling budget of *n*_samples_ evaluations per example (L1 path regularization set to num_features(64) to stabilize local fits in the high-dimensional setting). For modality-level importance, we report the sum of absolute SHAP across each modality’s feature dimensions and add the absolute SHAP of that modality’s prediction and uncertainty inputs; percentages are normalized so that the nine components (features/pred/var for each modality) sum to 100%. For feature-level summaries, we rank features within each modality by mean ∣SHAP∣ across test examples. Because WavLM embedding dimensions are latent coordinates without direct semantic meaning, we list their indices for completeness but do not seek clinical interpretations of individual dimensions.

### Statistics and reproducibility

All statistical analyses were conducted using Python (version 3.11.7) with standard scientific libraries such as SciPy, NumPy, and statsmodels. Performance metrics for classification tasks, including accuracy, sensitivity, specificity, PPV, NPV, AUROC, and Brier score, were reported with 95% confidence intervals (CIs), computed via bootstrapped resampling with replacement (*n* = 1000).

To compare model performance across test cohorts (Balanced Test Data, External Evaluation (Supervised), and External Evaluation (Unsupervised)) and against clinician ratings, we applied the non-parametric Mann–Whitney U test on the bootstrapped performance distributions. This test was selected because it does not assume normality and is appropriate for comparing independent, resampled metric distributions. To evaluate potential demographic biases in model performance by sex and race, we used Z-tests for proportions, applied only when the sample size met the standard assumptions (*np*≥5 and *n*(1 − *p*) ≥ 5). For comparisons involving categorical variables such as age groups or Hoehn and Yahr PD stages, we applied chi-square tests of independence. Since the expected cell frequency assumption (≥5) was violated in the PD-stage-based analysis, we used a Monte Carlo simulation with 10,000 replications to estimate *p*-values for the chi-square statistic, thereby ensuring valid inference in the presence of sparse data. Since we conducted multiple statistical tests on the same model predictions, which inflates the risk of Type I error, we applied a multivariable logistic regression analysis followed by Benjamini-Hochberg False Discovery Rate (FDR) correction to adjust the resulting *p*-values.

For assessing model calibration, Brier scores were computed, and calibration curves were generated by plotting the predicted PD probabilities against the observed outcome frequencies across bins. To assess inter-rater agreement among PARK predictions, clinician annotations, and ground-truth labels, we computed Cohen’s *κ* coefficient. In the usability study involving the Likert-scale responses (treated as ordinal or non-normally distributed continuous data), comparisons between demographic subgroups were conducted using the Mann–Whitney U test with FDR correction applied using the Benjamini-Hochberg procedure. All statistical significance tests were evaluated at a threshold of *p* < 0.05 after FDR correction, unless otherwise specified.

Each recording session was treated as an independent sample. When participants contributed multiple sessions across time, each session was analyzed separately but linked to the most recent clinical diagnosis available prior to that recording. Sample sizes for each analysis correspond to the number of participants or recording sessions included in the relevant dataset split, as detailed in the Results sections. No statistical methods were used to predetermine sample size.

### Ethics statement

This study utilized data from eight independent research protocols, each reviewed and approved by the Institutional Review Board (IRB) of the University of Rochester or the University of Rochester Medical Center (see Table 1 for study titles and corresponding protocol numbers). Each of the eight studies authorized the study team to analyze the data for research. Because this paper presents a secondary analysis of data obtained under those existing approved protocols, separate IRB approval for the present analysis was not required.

Eligible participants provided informed consent electronically via IRB-approved eConsent forms, administered either through REDCap or the study website (https://parktest.net)^26^. Ethical guidelines were followed to conduct all experiments and analyses presented in this manuscript.

## Results

### Study participants

A total of 1947 individuals are enrolled in this study. Of these, 82 do not contribute usable data due to corrupted video recordings and are excluded, leaving a final cohort of 1865 participants. Of these, 670 individuals (35.9%) have PD, including 380 with clinically confirmed diagnoses and 290 who self-report having PD. The cohort is demographically diverse (Fig. 1b), with 10.8% (*n* = 202) identifying as Non-white and 52.3% (*n* = 976) as female. In addition, the cohort includes 167 known carriers of the LRRK2 G2019S variant, of whom 40 have already been diagnosed with PD. Participants represent 36 countries (Fig. 1c) and range in age from 18 to 93 years (median = 63). Among those with a documented PD stage (Hoehn and Yahr scale) (*n* = 211), 142 (21.2%) have mild to moderate symptoms (stages 1–2), and 67 (10.0%) have moderate impairment (stage 3). Note that demographic information and country of residence are self-reported and occasionally missing.

Among the 1865 participants with usable data, 915 (300, 32.8% with PD) have completed all three standardized tasks and provided analyzable data across all task modalities. The remaining 950 either have skipped one or more tasks or have recordings that failed automated quality checks. Participants from SuperPD, ROOSTER-PD, Cluster-PD, and ROUTE-PD often contribute data across multiple sessions, where each session is defined as the three task recordings completed together. While sessions with missing tasks are used to train the task-specific models (if participants are included in the training set), only sessions with complete data are used to train (799 sessions) and evaluate (320 sessions) the UFNet model.

### Predictive performance

**Fig. 2:**
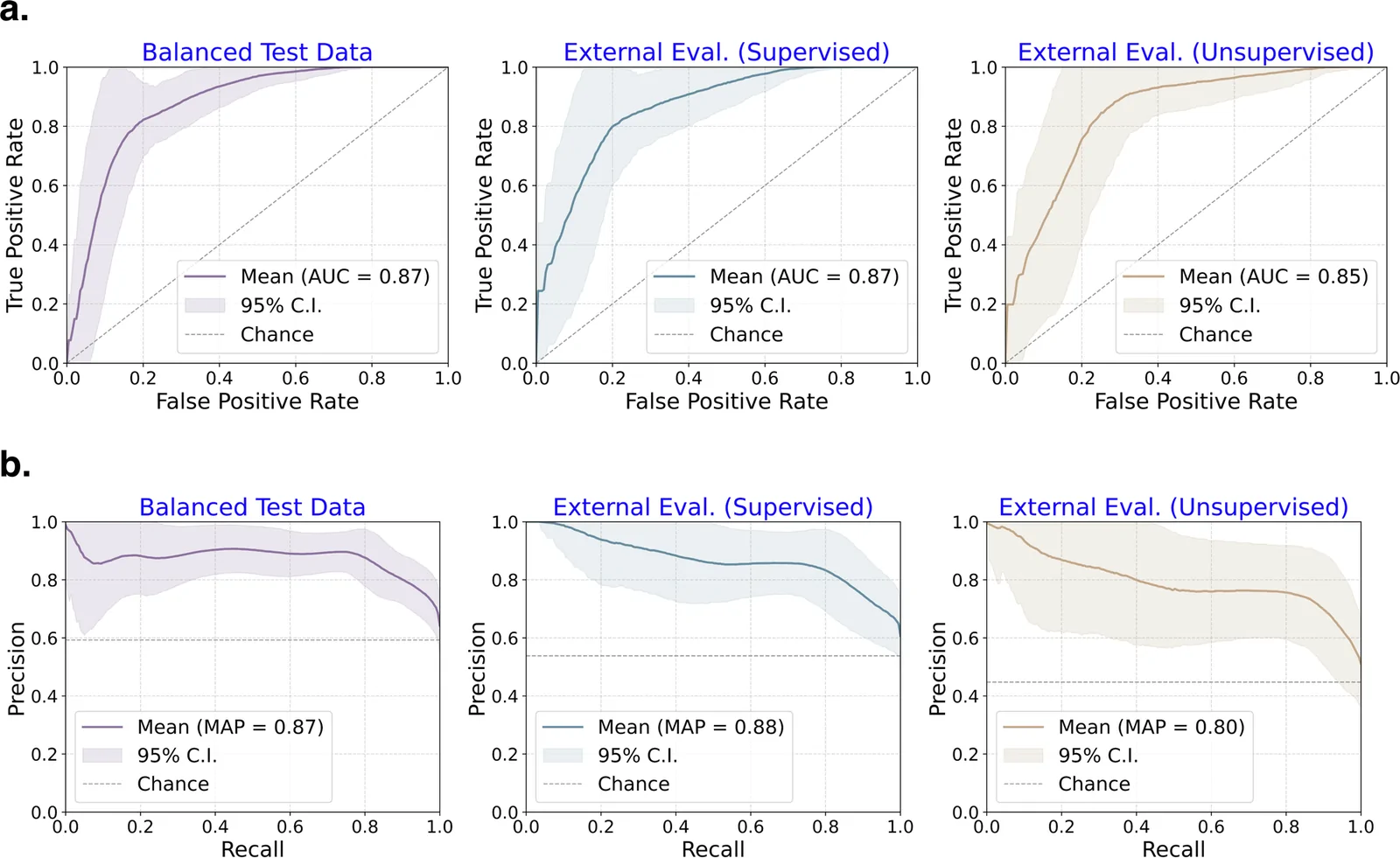
Predictive performance of the PARK tool. **a** Receiver operating characteristic (ROC) curves and **b** precision-recall curves demonstrate strong discriminative performance. The mean area under the ROC curve (AUROC) ranges from 0.85 to 0.87, and the mean average precision (MAP) ranges from 0.80 to 0.88 across the three evaluation cohorts. Models are evaluated on *N* = 124, *N* = 87, and *N* = 66 participants in the Balanced Test Data, External Evaluation (supervised), and External Evaluation (unsupervised) cohorts, respectively.

PARK demonstrates consistent performance in distinguishing individuals with and without PD across three evaluation settings (Table 2). Sensitivity remains stable, achieving 86.5 ± 6.8% (95% CI (confidence interval)) on the Balanced Test Data cohort and 85.7 ± 9.8% on the External Evaluation (supervised) cohort. However, both sensitivity and positive predictive value (PPV) are lower in the External Evaluation (unsupervised) cohort (83.3 ± 13.7% and 75.8 ± 14.3%, respectively). Pairwise statistical comparisons reveal that most of these differences are significant. Specifically, PPV in External Evaluation (unsupervised) is significantly lower than in both the Balanced Test Data and External Evaluation (supervised) cohorts, and sensitivity is significantly lower compared to Balanced Test Data (*p* < 0.001 for all three comparisons; Mann–Whitney U test). No significant difference in sensitivity is observed between External Evaluation (supervised) and External Evaluation (unsupervised) (*p* = 0.38). Notably, the model achieves its highest specificity and negative predictive value (NPV) when evaluated on unsupervised data (External Evaluation (unsupervised) cohort). We show the precision-recall and receiver operating characteristic (ROC) curves in Fig. 2.

**Table 2:** Summary of classification metrics

Test Set	Accuracy	Specificity	Sensitivity	PPV	NPV	F1 Score
Balanced Test Data (*n* = 162)	80.2 ± 6.2	71.2 ± 10.9	86.5 ± 6.8	81.4 ± 7.7	78.3 ± 10.6	83.8 ± 5.6
External Eval. (supervised) (*n* = 91)	80.2 ± 7.9	73.8 ± 12.8	85.7 ± 9.8	79.2 ± 10.8	81.6 ± 12.2	82.4 ± 8.0
External Eval. (unsupervised) (*n* = 67)	80.6 ± 9.2	78.4 ± 12.7	83.3 ± 13.7	75.8 ± 14.3	85.3 ± 12.2	79.4 ± 11.0

Metrics across internal (Balanced Test Data) and external evaluation cohorts (External Evaluation (supervised) and External Evaluation (unsupervised)) demonstrate consistently high accuracy, sensitivity, and *F*_1_ scores. All values are reported as percentages (%), and *n* denotes the number of independent sessions. Results are reported as 95% confidence intervals (mean ± 1.96 × standard error).

**Fig. 3:**
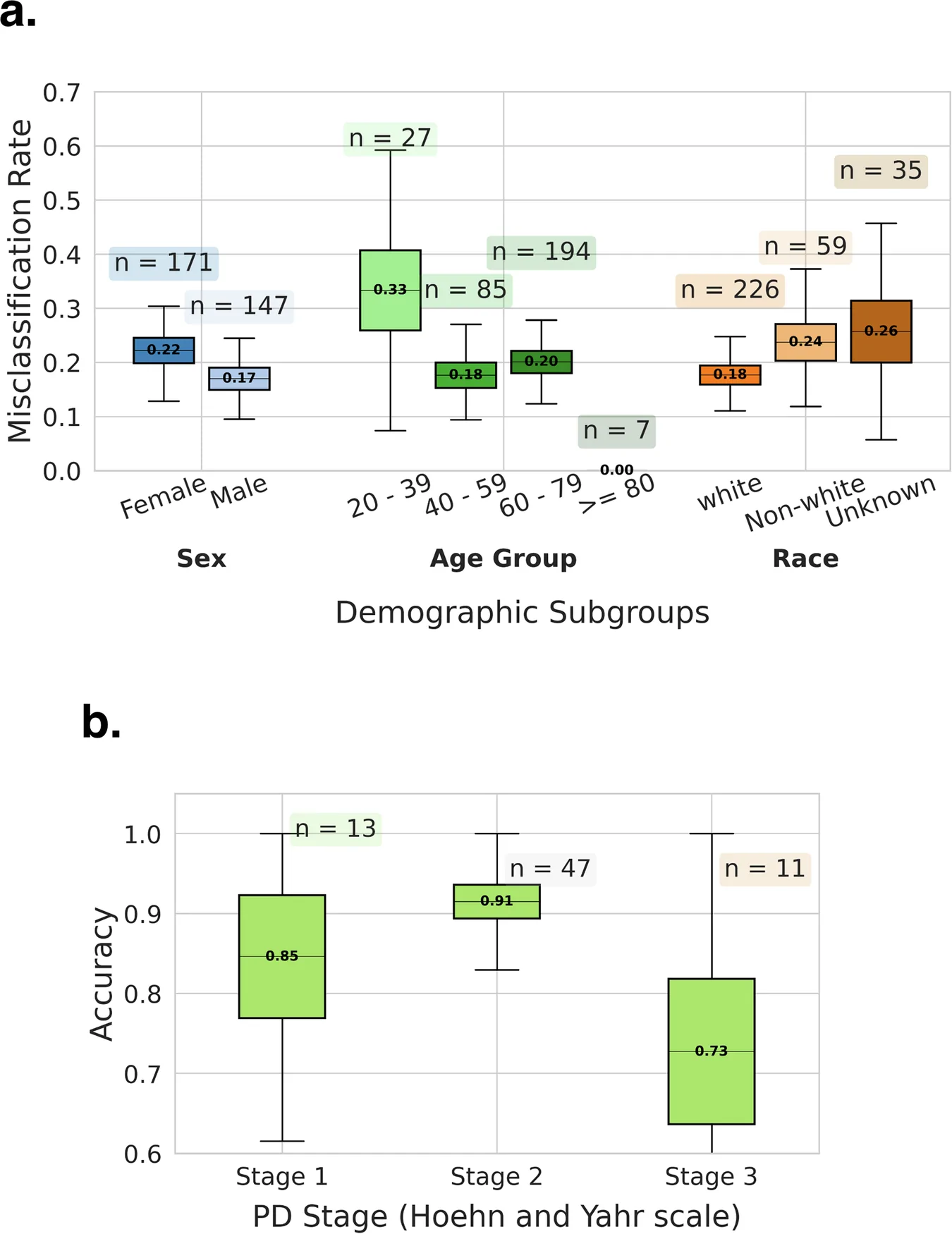
Performance across subgroups. **a** Misclassification rates stratified by sex, age, and race; **b** Accuracy across PD stages based on the Hoehn and Yahr scale, with the highest performance observed in individuals at stage 2. While some variability is noted, no subgroup demonstrates disproportionately elevated error rates. *n* represents the number of sessions included in each subgroup. Boxplots show the distribution of values for each subgroup; the center line indicates the median; the box spans the interquartile range (IQR); and the whiskers extend to 1.5  × IQR from the first and third quartiles.

Among the 320 evaluation sessions, 71 are from individuals with documented PD stage (Hoehn and Yahr scale). PARK achieves mean predictive accuracies of 84.2% (65.3−100%), 91.4% (83.4−99.4%), and 73.0% (46.8−99.2%) for participants at stage 1 (mild symptoms), stage 2 (mild-to-moderate symptoms), and stage 3 (moderate symptoms), respectively (Fig. 3). Although the model generally performs better for participants with mild and mild-to-moderate symptoms, we do not detect a statistically significant difference in predictive accuracy across stages (test statistic: *χ*^2^ = 2.94, *p* = 0.157; Monte Carlo-simulated chi-square test).

We further assess model performance across demographic subgroups, combining data from all three evaluation cohorts. The PARK tool shows slightly lower accuracy for under-represented groups, including females (77.8 ± 6.7%) and Non-white individuals (76.3 ± 11.3%). However, no subgroup based on sex or race exhibits a significantly higher misclassification rate (*p* = 0.245 for sex and *p* = 0.293 for race; using Z-tests for proportions). Additionally, a chi-square test finds no significant variation in error rates across age groups (*p* = 0.206).

**Fig. 4:**
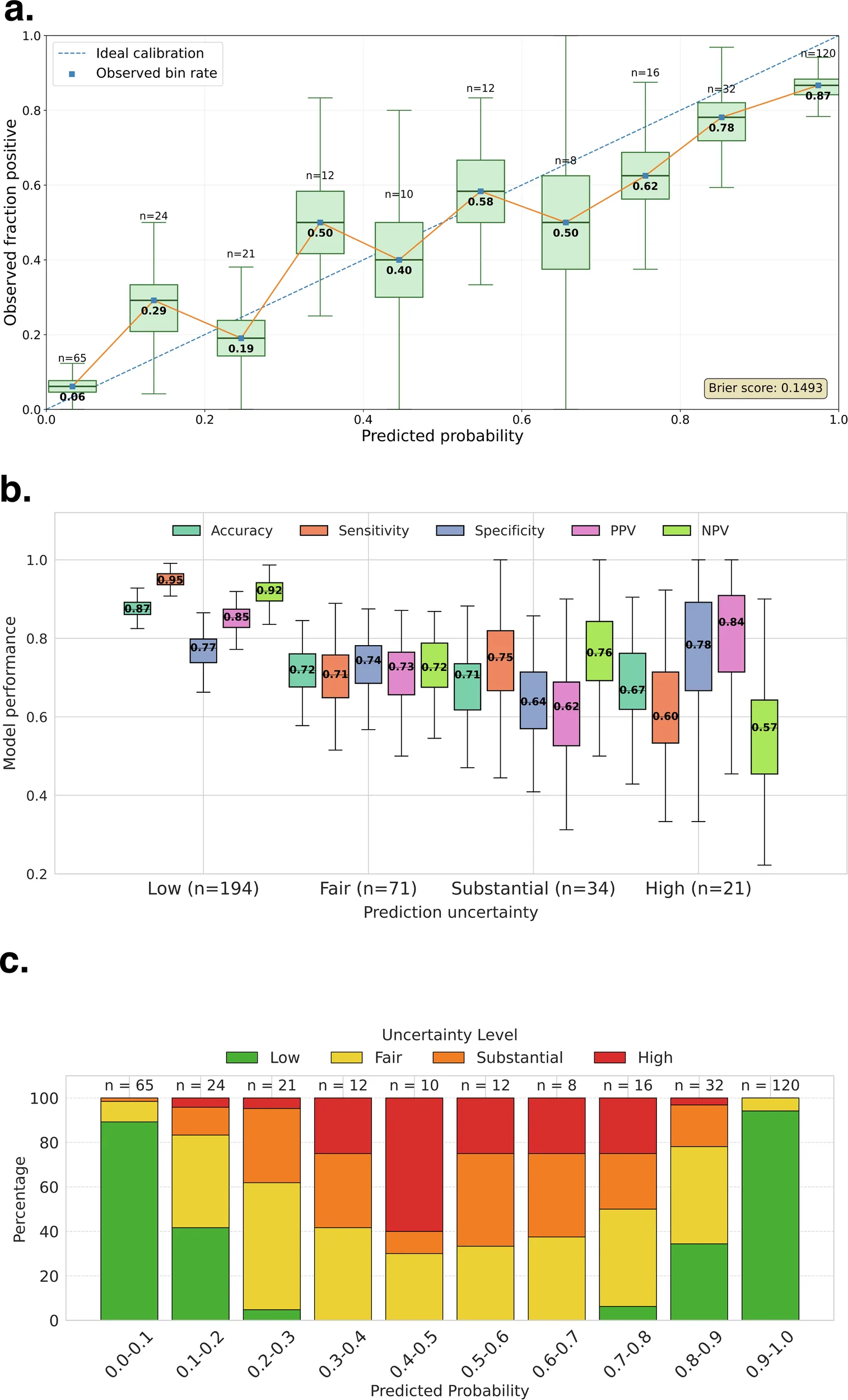
Evaluation of model uncertainty. **a** Calibration curve showing alignment between predicted probabilities and observed outcomes (Brier Score = 0.1493), indicating reasonable model calibration. **b** Model performance across different levels of prediction uncertainty. **c** Relation between predicted probability and prediction uncertainty. Boxplots in **a**, **b** show the distribution of values for each subgroup (*n* represents the number of sessions included in the subgroup); the center line indicates the median; the box spans the interquartile range (IQR); and the whiskers extend to 1.5  × IQR from the first and third quartiles.

Beyond binary classification, the PARK tool provides a continuous score ranging from 0 to 1 that can be interpreted as the likelihood of PD. We evaluate the calibration of these likelihood scores using calibration curves and the Brier score^49^. PARK achieves a Brier score of 0.149, indicating moderate calibration. Likelihood estimates below 0.10 or above 0.80 are well aligned with true outcome probabilities (Fig. 4). However, calibration is less reliable in the mid-range (0.3–0.6), where predicted scores deviate more from observed probabilities.

We further examine how predictive uncertainty relates to model performance and to potential confidence in clinical decisions. Based on the premise that uncertain predictions exhibit greater variability across multiple MC dropout rounds, we stratify the test data into four uncertainty bins–Low, Fair, Substantial, and High–according to the standard deviation of model predictions (Low: 0–1.5%, Fair: 1.5–3.0%, Substantial: 3.0–4.5%, High:  > 4.5%). We then compute key performance metrics (accuracy, sensitivity, specificity, PPV, and NPV) within each group. Model performance is the highest in the Low-uncertainty group (Accuracy = 87.4 ± 4.7%, sensitivity = 94.9 ± 3.9%, specificity = 77.1 ± 9.3%) and declines with increasing uncertainty, indicating that predictive uncertainty serves as a meaningful proxy for diagnostic confidence. In the High-uncertainty group (*n* = 21), accuracy drops to 66.7%, reflecting reduced reliability.

Finally, to investigate the source of the suboptimal calibration observed in predictions with mid-range PD likelihood, we examine the relationship between the predicted probability and the corresponding uncertainty. Predictions with extreme probabilities (near 0 or 1) exhibited low uncertainty, indicating high model confidence. In contrast, mid-range probabilities are associated with substantially higher uncertainty. For example, within the 0.3–0.4 probability range, none of the predictions fall under the Low uncertainty category, and 58% are classified as Substantial or High uncertainty.

### Consistency with clinician evaluation

Against ground truth screening obtained via majority voting of three expert neurologist judgments, PARK achieves an accuracy of 83.7 ± 13.2% and sensitivity of 86.7 ± 14.3%. The three clinicians achieve accuracies of 80.2 ± 14.1%, 86.9 ± 12.2%, and 76.5 ± 14.7%, with corresponding sensitivities of 95.6 ± 8.7%, 95.6 ± 8.8%, and 77.2 ± 17.0%. Notably, the two clinicians with the highest sensitivity have lower specificity (37.7 ± 33.9% and 63.0 ± 12.2%), reflecting a trade-off favoring recall. In contrast, PARK exhibits more balanced performance across metrics (Table 3). Pairwise Mann–Whitney U Test of bootstrapped distributions across the three clinicians and the clinician majority vote reveals that PARK’s sensitivity, NPV, and *F*_1_ score are significantly lower than those of at least one clinician (FDR-adjusted *p* < 0.05), with NPV also significantly lower than the majority vote (*p* < 0.01). However, PARK’s accuracy, specificity, and PPV do not differ significantly from those of any individual clinician or the majority vote (FDR-adjusted *p *≥ 0.527).

**Table 3:** Comparison of PARK with specialist evaluations

	Accuracy	Specificity	Sensitivity	PPV	NPV	*F*_1_ Score
Specialist 1	80.2% ± 14.1%	37.7% ± 33.9%	95.6% ± 8.7%	80.9% ± 14.7%	75.2% ± 50.2%	87.4% ± 9.9%
Specialist 2	86.9% ± 12.2%	63.0% ± 35.8%	95.6% ± 8.8%	87.6% ± 13.3%	84.1% ± 33.1%	91.2% ± 8.7%
Specialist 3	76.5% ± 14.7%	75.1% ± 31.3%	77.2% ± 17.0%	89.2% ± 14.2%	54.8% ± 29.5%	82.4% ± 12.5%
PARK	83.7% ± 13.2%	75.2% ± 31.7%	86.7% ± 14.3%	90.7% ± 12.2%	67.4% ± 32.7%	88.4% ± 10.2%

Classification performance of PARK and three PD specialists on *n* = 30 individuals from the evaluation cohorts with confirmed diagnoses. PARK achieves balanced sensitivity (86.7 ± 14.3%) and specificity (75.2 ± 31.7%), while specialists show higher sensitivity but lower specificity. Results are reported as 95% confidence intervals (mean ± 1.96 × standard error).

**Fig. 5:**
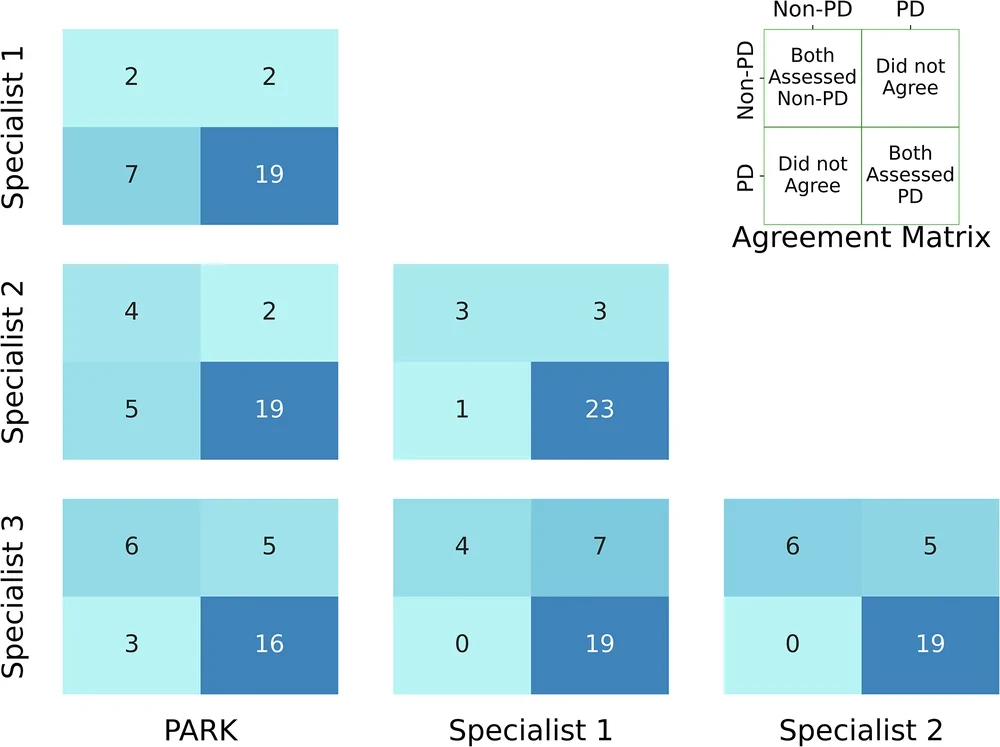
Agreement between specialists and PARK. Confusion matrices show prediction patterns among the specialist-specialist and PARK-specialist pairs.

Pairwise confusion matrices (Fig. 5) reveal stronger consensus among the clinicians and PARK for positive predictions, particularly when PARK identifies PD. This observation is consistent with the high sensitivity and PPV observed across both PARK and the clinicians. Inter-rater agreement analysis further contextualizes this alignment. PARK achieves the highest concordance with ground-truth diagnoses, with a Cohen’s *κ* of 0.590 and an overall agreement of 83.3%. Clinician-clinician agreement is similar (*κ* = 0.516, 82.2%). In contrast, alignment between a single clinician and ground truth (*κ* = 0.496, 81.1%) and between a single clinician and PARK (*κ* = 0.313, 73.3%) is lower.

### User-centric evaluation of PARK

**Fig. 6:**
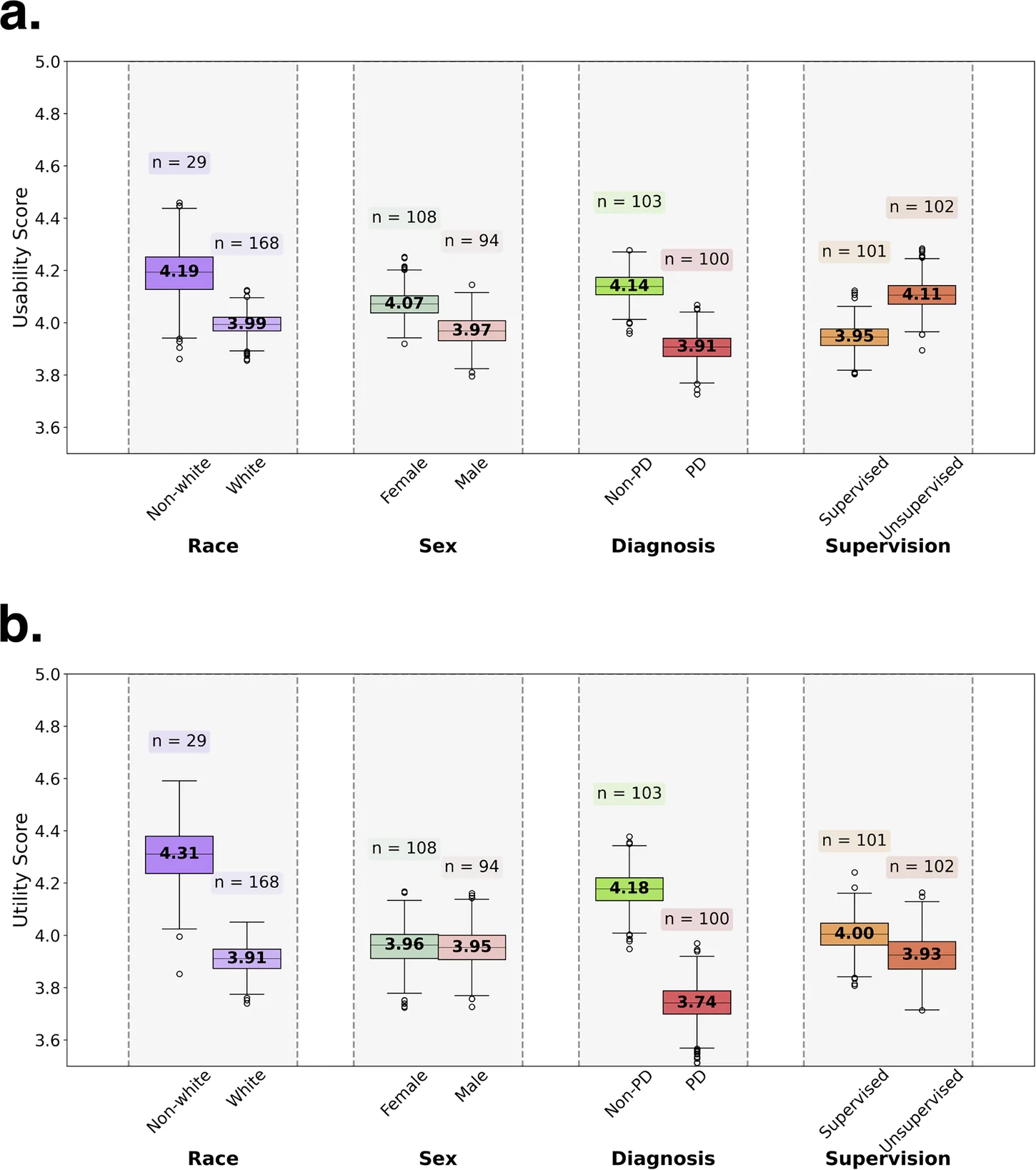
Usability and Utility of the PARK screening tool. Questions are answered on a five-point Likert scale (strongly disagree, disagree, neutral, agree, strongly agree). Higher scores indicate more favorable responses. **a** Usability scores are consistently high across demographic subgroups. **b** Perceived utility of PARK is similarly favorable across most subgroups. *n* represents the number of survey sessions included in each subgroup. Boxplots show the distribution of values for each subgroup; the center line indicates the median; the box spans the interquartile range (IQR); the whiskers extend to 1.5  × IQR from the first and third quartiles; and points beyond the whiskers represent outliers.

The usability of the PARK tool is evaluated using Likert-scale items that assess ease of use and user interaction, including the standard 10-item System Usability Scale (SUS)^25^. The average SUS scores are 70.8 ± 1.3% for the supervised cohort and 75.5 ± 1.6% for the unsupervised cohort, indicating acceptable usability across both environments. Overall usability ratings are high (mean score 4.03/5.00) and consistent across demographic groups, PD status, and supervision level, suggesting broad accessibility. Similarly, perceived utility–whether PARK’s assessments and resources were understandable, informative, and comprehensive–receives favorable feedback (mean score 3.96/5.00) with consistent ratings across sex and supervision subgroups (see Fig. 6 for details).

**Fig. 7:**
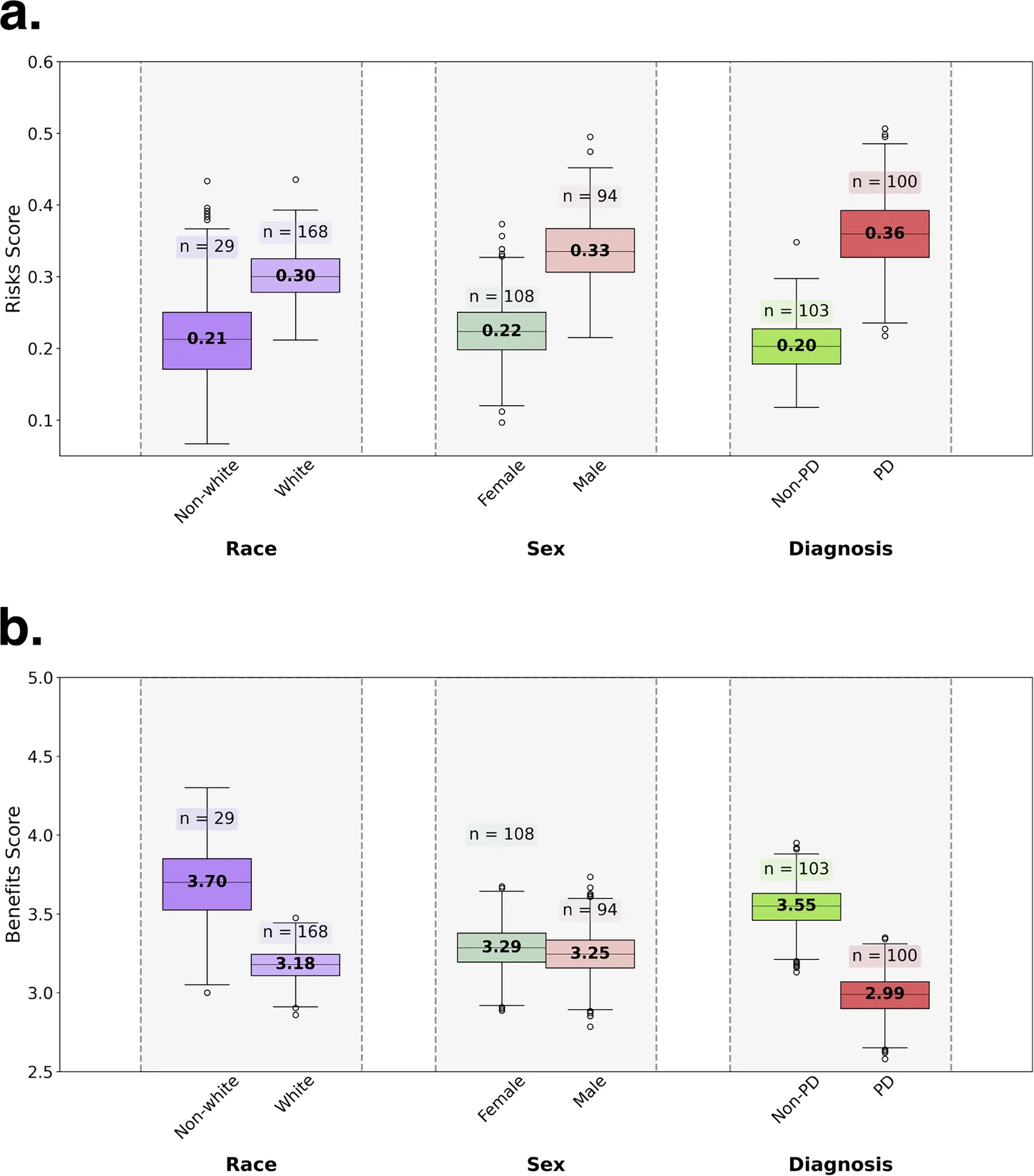
Benefits and Risks of using the PARK screening tool across demographic subgroups. Questions about perceived risks are binary (e.g., whether PARK caused anxiety or its responses were potentially harmful), whereas questions about benefits are rated on a five-point Likert scale (strongly disagree, disagree, neutral, agree, strongly agree). A lower risk score is better, while higher benefit scores indicate more favorable responses. **a** Perceived risks are low overall but elevated among individuals with PD. **b** Perceived benefits are rated more favorably by Non-white and non-PD users. *n* represents the number of survey sessions included in each subgroup. Boxplots show the distribution of values for each subgroup; the center line indicates the median; the box spans the interquartile range (IQR); the whiskers extend to 1.5  × IQR from the first and third quartiles; and points beyond the whiskers represent outliers.

**Fig. 8:**
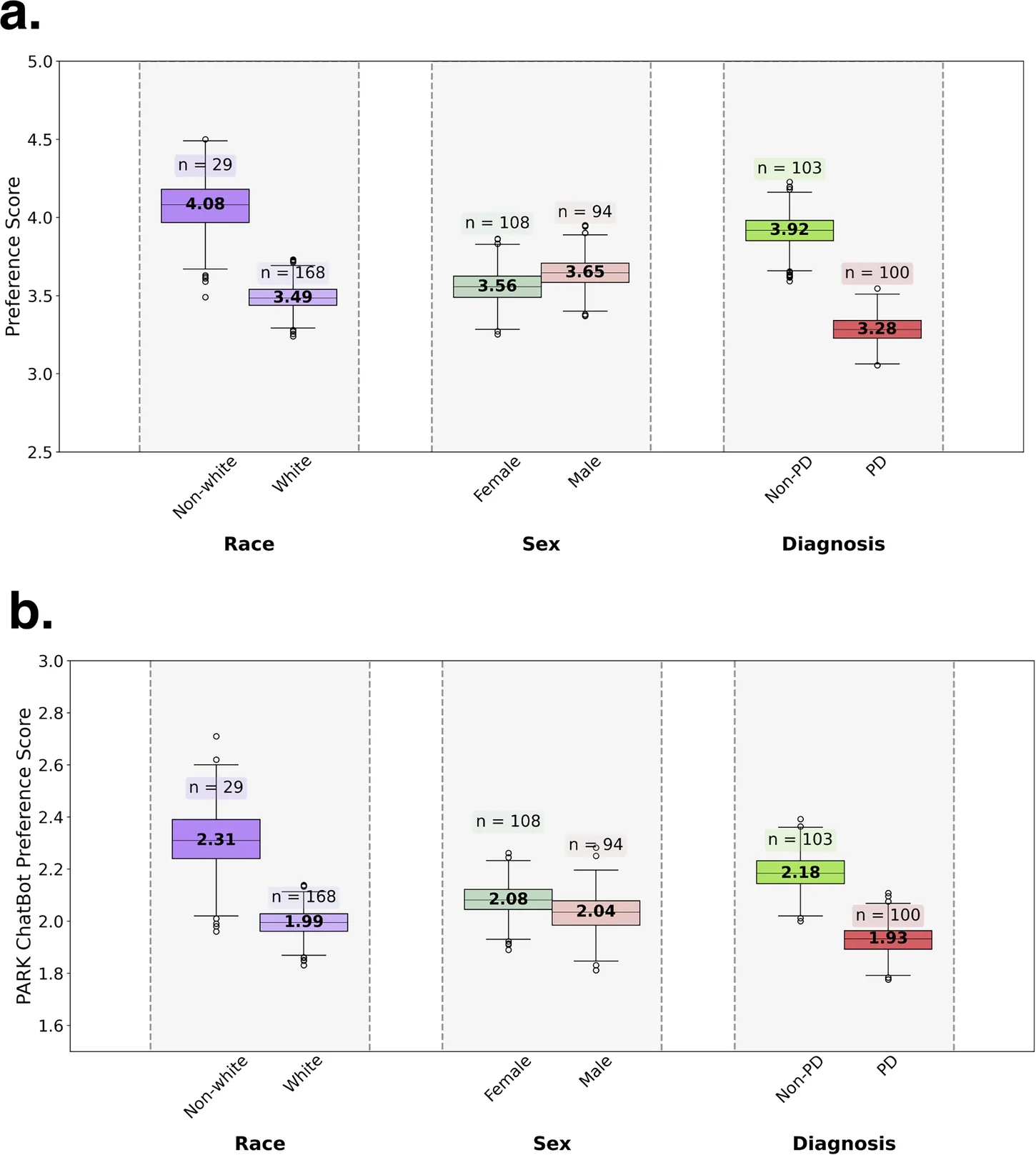
User preferences for the PARK screening tool across demographic subgroups. Questions about the overall preference for the PARK tool use a five-point Likert scale (strongly disagree, disagree, neutral, agree, strongly agree), whereas preferences for the chatbot are rated on a three-point Likert scale (often, sometimes, never). Higher scores indicate more favorable responses. **a** Preference for PARK over traditional care is higher among Non-white and non-PD participants. **b** Willingness to interact with the PARK chatbot is moderate overall, with higher favorability among Non-white and non-PD groups. *n* represents the number of survey sessions included in each subgroup. Boxplots show the distribution of values for each subgroup; the center line indicates the median; the box spans the interquartile range (IQR); the whiskers extend to 1.5  × IQR from the first and third quartiles; and points beyond the whiskers represent outliers.

Participants in the unsupervised cohort also rate the perceived risks and benefits of using PARK (Fig. 7) and their preferences for the tool and the integrated chatbot (Fig. 8). On average, users rate the tool as low risk (0.28/1.00) and moderately beneficial (3.27/5.00). Participants express a general preference for using PARK (average score 3.6/5.00) over in-person clinical appointments when considering specific factors such as accessibility (4.14/5.00), convenience (4.21/5.00), comfort (2.91/5.00), privacy (3.53/5.00), and financial burden (3.20/5.00). However, preferences regarding the LLM-powered chatbot are mixed (average score 2.05/3.00).

User ratings do not differ significantly by sex across any of the six evaluated dimensions (*p* = 0.605, 0.662, 0.662, 0.113, 0.955, 0.662, respectively, using the Mann–Whitney U test). However, perceived risk is notably elevated among male participants (*p* = 0.113). Non-white participants express more favorable views of PARK across most categories, with statistically significant differences observed in usability, perceived utility, preference for PARK over clinical visits, perceived benefits, and willingness to use the chatbot (*p* = 0.042, 0.003, 0.002, 0.016, and 0.002, respectively). Similar trends are observed among participants without PD, who rate PARK more favorably on usability, perceived utility, preference for PARK over clinical visits, perceived benefits, and willingness to use the chatbot (*p *≤ 0.002 for all cases). However, participants with PD perceive the risks to be higher than participants without PD (*p* = 0.023).

Finally, participants in the unsupervised Validation Study-2 rate PARK as more usable than those in the supervised Validation Study-1 (*p* = 0.009), while perceived utility does not differ significantly by study supervision status (*p* = 0.582). Note that all the above-reported *p* values are FDR-adjusted using the Benjamini-Hochberg (BH) procedure^51^.

### Investigation of model errors

**Fig. 9:**
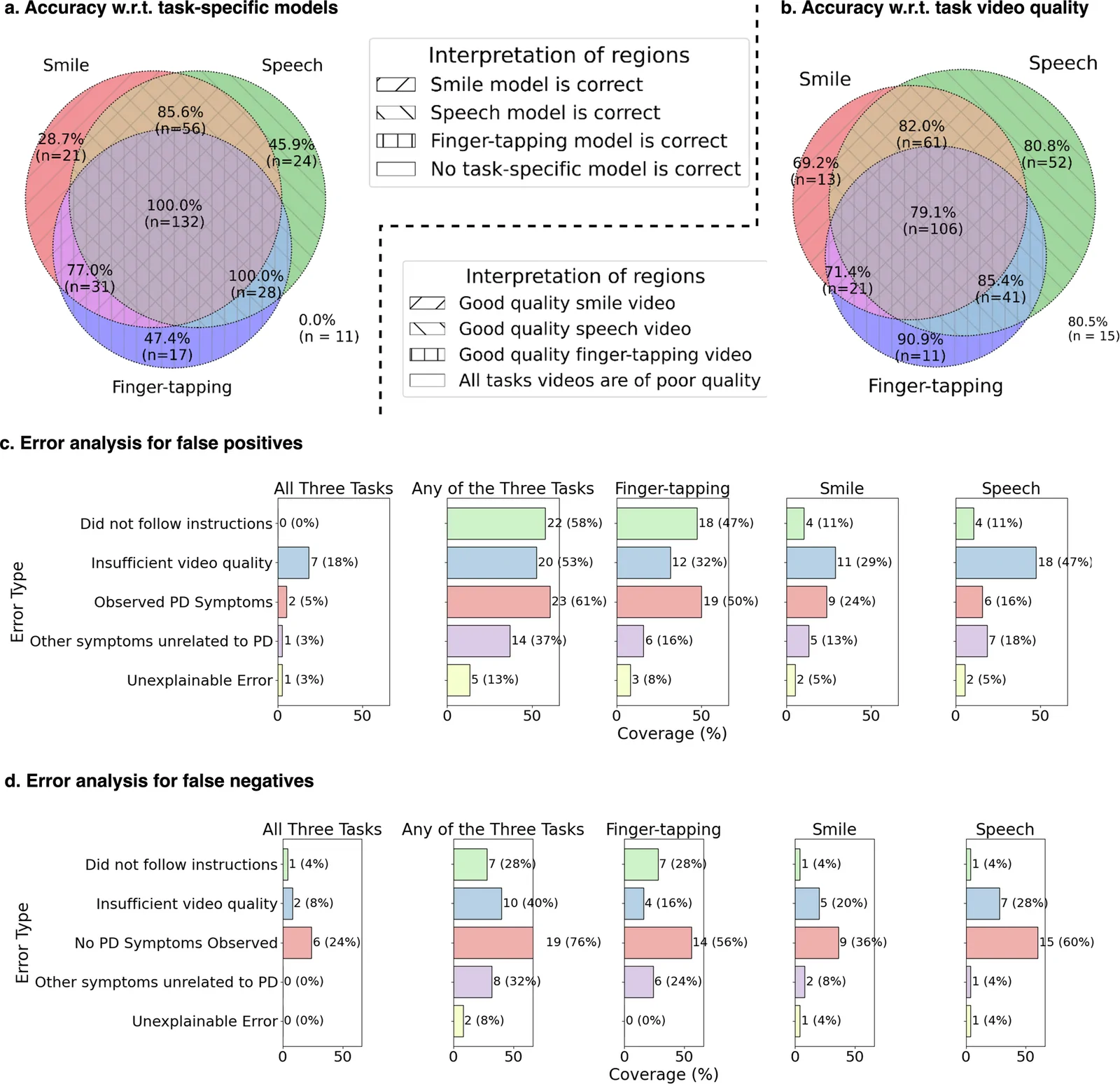
Accuracy decomposition and error analysis of the PARK tool across tasks and video conditions. **a** Venn diagram annotated with PARK's prediction accuracy (%) and number of corresponding sessions (n) relative to the accuracy of task-specific models. Each region enclosed by a circle represents cases in which one or more task-specific models (smile, speech, or finger-tapping) correctly predict the PD status. Notably, PARK achieves 100% accuracy when all three task-specific models are correct, and an accuracy of 77.0–100.0% even when one of the models is incorrect. **b** Corresponding analysis based on task video quality, showing that PARK maintains consistent accuracy across varying levels of video quality. **c** Error analysis of false positives suggests that failure to follow instructions, insufficient video quality, and observed PD symptoms (particularly in the finger-tapping task) despite negative ground truth, may contribute to erroneous positive predictions. **d** False negatives are most commonly associated with the absence of visible PD symptoms, particularly in the speech and finger-tapping tasks, which together account for 56–60% of such errors.

PARK demonstrates consistent ability to integrate signals from the task-specific models (Fig. 9a). As expected, PARK achieves 100 ± 0.0% accuracy when all three task-specific models correctly identify PD status (*n* = 132). However, the tool also maintains high performance when one task-specific model is incorrect. For instance, PARK maintains 100 ± 0.0% accuracy when both the finger-tapping and speech models are correct (*n* = 28), and 85.6 ± 9.0% accuracy when the smile and speech models are correct (*n* = 56). Speech and finger-tapping models are particularly influential–PARK achieves 45.9 ± 20.4% accuracy (*n* = 24) when only the speech model is correct (and the other two models are wrong), and 47.4 ± 24.3% accuracy (*n* = 17) when only the finger-tapping model is correct.

PARK also performs reliably despite variability in task video quality (Fig. 9b). PARK maintains 80.5 ± 19.5% accuracy even when none of the task videos are labeled by human annotators as good quality (*n* = 15), which is comparable to its accuracy when all three task videos are good quality (79.1 ± 7.7%, *n* = 106). Note that videos with automatically detected trivial quality issues are excluded from analysis.

To understand model errors, two doctoral students with 3–5 years of experience in PD video analysis have manually reviewed all sessions in which PARK produced false positives or false negatives. They label each task video with possible contributing factors: failure to follow instructions, poor video quality, observable Parkinsonian symptoms in individuals not diagnosed with PD, other non-PD symptoms that may have confused the model, or lack of visible PD symptoms in diagnosed individuals. If none of these factors applied, the video is marked as an unexplained error.

Among the 38 false-positive sessions, a common factor is poor video quality: 20 sessions (53%) have at least one low-quality task video, and 7 (18%) have quality issues across all three tasks. Annotators have observed Parkinsonian-like symptoms in 23 sessions (61%), with 2 (5%) showing such symptoms across all three tasks. Noncompliance with task instructions is also prevalent, noted in 22 sessions (58%). Only one session (3%) is marked as an unexplained false positive (with no apparent contribution factor for model error).

Among the 25 false-negative cases, the most frequent issue is the absence of observable PD symptoms, noted in 76% of sessions (across one or more tasks) and in 24% of sessions across all tasks. Poor video quality is also associated with false negatives: 2 sessions (8%) had inadequate quality across all tasks, and 10 sessions (40%) had issues in at least one task.

### Model explainability

**Fig. 10:**
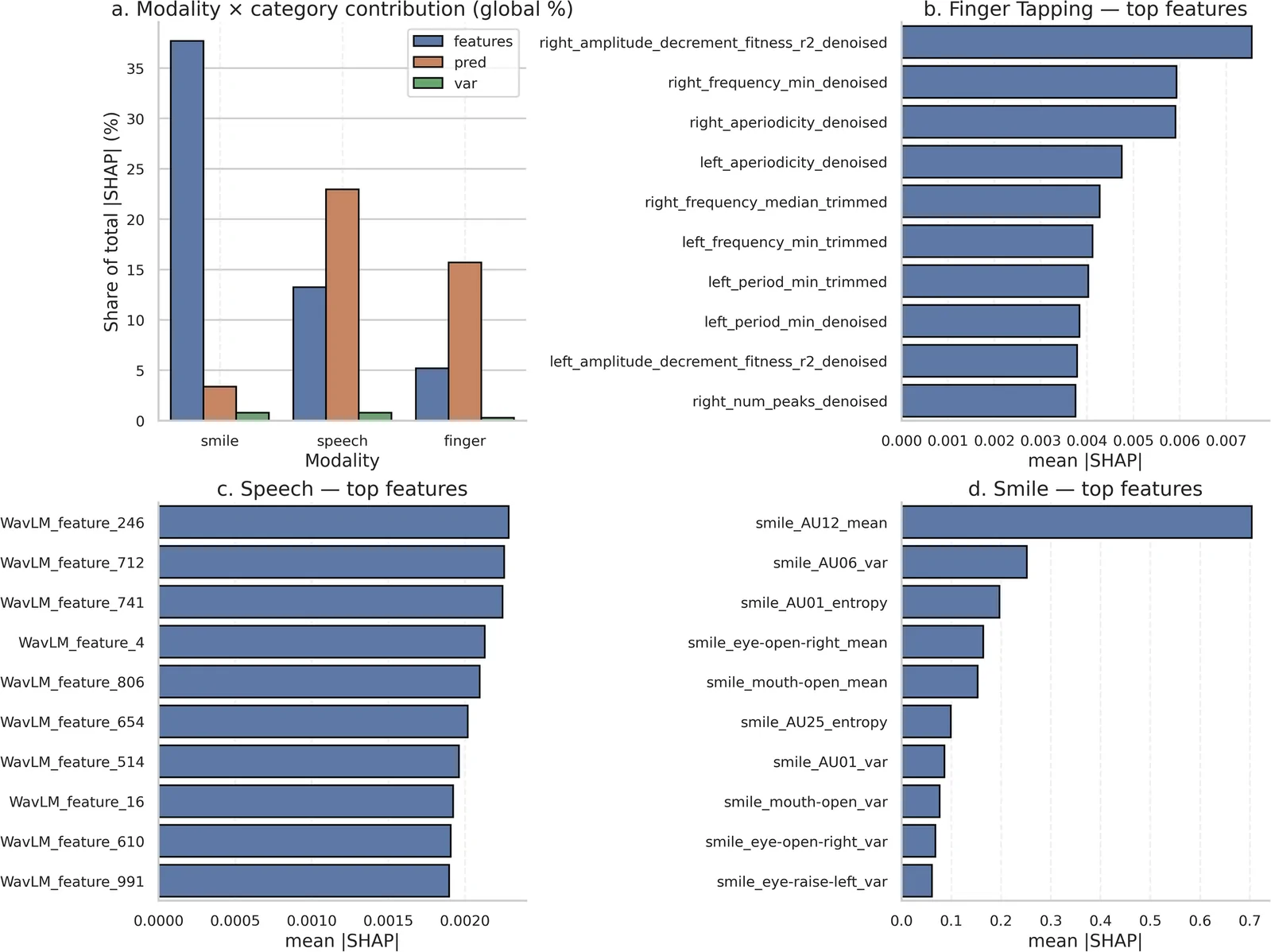
SHAP analysis on UFNet predictions. **a** Relative contributions of different information sources (low-level features, unimodal prediction scores, and prediction uncertainty) for each modality (finger tapping, speech, and smile). Each bar represents the proportion of total explanatory weight, normalized to sum to 100%, showing which modalities and feature types most strongly influence UFNet’s outputs. **b**–**d** Within-modality rankings of the most influential low-level features. All SHAP values are computed on the combined test set using a k-means background derived from the training data.

To investigate which modalities and features guide UFNet’s predictions, we perform post-hoc interpretation using SHapley Additive Explanations (SHAP^50^). UFNet integrates both low-level task-specific features and high-level predictive information, captured as the mean and variance of MC dropout predictions. Our analysis reveals that the smile and speech modalities contribute most strongly to model decisions (41.8% and 37.0%, respectively). Interestingly, the model relies primarily on high-level predictive information for the speech and finger-tapping tasks (Fig. 10a), but emphasizes low-level features when evaluating smile videos, with these features accounting for 36% of SHAP weights for the final prediction. This pattern suggests that, although smile-only predictions are less reliable, their low-level features still provide complementary information when combined with high-level predictive signals from the other tasks.

Among the low-level features (Fig. 10b–d), the most influential drivers for the finger-tapping task are a decrease in amplitude, tapping frequency (taps per second), and tapping regularity, the latter quantified using an aperiodicity metric^18^, for both hands. For the smile task, key contributors include the mean, variance, and entropy of several facial action units (AU12, AU06, and AU01). By contrast, the speech task relies on low-level deep representations extracted from the WavLM^46^ model, which are less readily interpretable.

## Discussion

In this study, we present PARK, an AI-powered screening tool for PD that leverages webcam-recorded videos of facial, motor, and speech tasks. Across three independent cohorts, including a demographically balanced test set and two real-world deployment settings, PARK demonstrates strong and consistent predictive performance. Notably, PARK achieves 80.2−80.6% accuracy, 75.8−81.4% PPV, 78.3−85.3% NPV, 79.4−83.8% *F*_1_-score, and 85.0−87.0% AUROC, while maintaining robustness across demographic subgroups and video quality conditions. In parallel, user-centric evaluations across supervised and unsupervised environments reveal high usability (SUS > 70), broad accessibility, and favorable perceptions across six dimensions, including perceived utility, risks vs. benefits, and preferences over traditional clinical care. These results position PARK as a scalable, user-friendly screening solution with the potential to extend access to neurological assessment in resource-limited or underserved settings. There is a nationwide shortage of clinical neurologists. The shortage is even more acute for neurologists skilled in the care of patients with movement disorders. One can imagine a patient with potential signs of PD, such as involuntary tremors, undergoing analysis with PARK, with triage decisions based on these results. For such individuals, PARK can serve as a first-line, accessible tool to help them recognize concerning symptoms and seek clinical evaluation sooner. Healthcare systems can reduce long-term economic costs by prioritizing screened individuals and delivering early clinical interventions that enable timely treatment.

From a diagnostic standpoint, PARK achieves high sensitivity (83.3−86.5%) and moderate specificity (71.2−78.4%) across diverse cohorts, suggesting utility for identifying individuals with PD without substantially increasing false positives. Nonetheless, false positives may lead to unnecessary anxiety, while false negatives may delay care–highlighting the importance of responsible result communication. To mitigate these risks, PARK incorporates uncertainty-aware prediction withholding, communicates results as probabilistic risk scores (rather than definitive diagnoses), and encourages users to consult medical professionals. These safeguards, combined with strong usability ratings and favorable perceptions among end users, support PARK’s safe deployment as a screening, not diagnostic, tool. Its ability to operate reliably across demographic subgroups and video quality conditions further underscores its readiness for equitable, real-world use.

We note that participants at more advanced Hoehn and Yahr stages often exhibit pronounced motor and speech impairments, which can make it challenging to complete standardized webcam-based tasks or maintain stable video capture, potentially introducing noise and reducing model accuracy. In addition, such individuals may require caregiver assistance or specialized clinical support-contexts for which PARK is not primarily designed. Our focus is on individuals in early- to mid-stage (stages 1–2), where timely screening can prompt clinical evaluation and intervention before substantial functional decline.

The observed relationship between predictive uncertainty, performance, and calibration (Fig. 6) carries important clinical implications. In PARK, low-uncertainty predictions correspond to high diagnostic accuracy, whereas mid-range predictions show elevated uncertainty and poorer calibration, reflecting the model’s reduced confidence in borderline cases. This behavior mirrors clinical reasoning, where ambiguous cases naturally warrant closer examination. In practice, such uncertainty estimates could serve as a built-in mechanism for confidence-aware decision support: low-uncertainty cases may be communicated with higher confidence, while high-uncertainty or mid-probability cases could be automatically flagged for clinician review, repeat assessment, or supplementary data collection. Incorporating such adaptive uncertainty thresholds into deployment would help balance automation with expert oversight, ensuring that AI-driven screening tools like PARK augment, rather than replace, clinician judgment in PD assessment.

Relative to prior efforts in PD detection using smartphone-based tasks^52,53^ or wearable and passive sensing approaches^54,55^, PARK emphasizes short, structured webcam tasks that are simple to perform at home and produce interpretable outputs. Although some studies^52,56^ have reported similar or superior performance with wearable sensors, such technologies often face greater logistical barriers to deployment in underserved communities. By contrast, PARK provides a low-friction, “visit-like” screening paradigm that is well suited for individuals with limited access to specialized PD care.

Despite recent advances in video understanding, including multimodal large language models (LLMs), these models may not be appropriate for our application. LLMs typically require substantial computational resources and are impractical for local deployment. Reliance on cloud-hosted models would also necessitate uploading patient videos, raising significant privacy concerns. In prior work on UFNet^24^, we compared this lightweight architecture to alternative modeling strategies and demonstrated its advantages in balancing performance, interpretability, and feasibility. This study focuses instead on rigorously evaluating UFNet’s performance across a demographically diverse cohort, as well as its user acceptance and clinical alignment.

PARK not only achieves accuracy, PPV, and *F*_1_ scores comparable to those of three movement disorder specialists (under the same *video-only* evaluation conditions), it demonstrates higher specificity (75.2 ± 31.7%) and the strongest agreement with ground truth diagnoses (Cohen’s *κ* = 0.590). Clinicians, by contrast, show higher sensitivity (77.2–95.6%) but lower specificity (37.7–75.1%), likely reflecting a recall-oriented strategy aimed at avoiding missed cases. Most discrepancies occur when clinicians predict PD and PARK do not (Fig. 5), highlighting this difference in diagnostic behavior. However, these comparisons should be interpreted with caution, as the video-only evaluation does not mirror real-world clinical diagnosis. Clinicians reviewed only three recorded videos of facial expression, finger tapping, and speech, and did not perform full clinical assessments due to logistical constraints. Their performance would have been stronger in a traditional in-person encounter. Moreover, the current comparison is limited by a small sample size (*n* = 30 sessions), rendering the estimates exploratory rather than definitive. A power analysis based on the McNemar framework indicates that approximately 775 samples would be required to demonstrate PARK’s non-inferiority to clinicians (non-inferiority margin = 3%, *α* = 0.025, power = 80%) under similar observed effect sizes. Nevertheless, the findings suggest that PARK can approximate clinician-level performance on focused video-based assessments while offering greater accessibility. Importantly, its decision threshold can be tuned to suit deployment needs–emphasizing sensitivity in low-access regions or prioritizing precision in overburdened systems–making it adaptable to both user preferences and clinical demands.

User feedback across two external evaluation studies demonstrates broad acceptability of the PARK tool, with high usability scores (mean SUS: 70.8 ± 1.3% when supervised, and 75.5 ± 1.6% when unsupervised) and favorable ratings across six experience dimensions. Notably, Non-white participants consistently rate PARK more positively than white participants in usability (FDR-adjusted *p* = 0.042), perceived utility (*p* = 0.003), benefits (*p* = 0.016), preference over clinical visits (*p* = 0.002), and willingness to engage with the chatbot (*p* = 0.002). This disparity may reflect more limited access to traditional neurological care among Non-white individuals, who may perceive PARK as a more viable or empowering alternative. In contrast, participants with PD, most of whom had received a diagnosis and were already connected to clinical care, tended to view the tool as slightly less useful and of significantly higher risk. These findings underscore the importance of considering lived experience and access to care when designing and deploying AI screening tools. PARK’s favorable reception among underserved groups suggests it may help reduce disparities in access to neurological assessments, particularly when thoughtfully integrated into existing care pathways. These psychological factors are crucial for adoption among the target population.

Participants’ open-ended feedback reveals a consistent and nuanced perspective on trust, usability, and the appropriate role of AI in healthcare. While users value PARK’s simplicity, clarity, and accessibility, most express that they would prefer clinician confirmation and would not rely solely on an AI-generated diagnosis. As one participant with PD notes, “*If a diagnosis of PD was being given through the PARK tool, it would be pretty rough for the patient-it would be important to have a medical professional reveal the diagnosis*.” reflecting understandable caution about automated health feedback. At the same time, many participants recognize PARK’s value as a low-friction entry point for early screening and guidance. One control participant describes it as “*fantastic for people without access to doctors and people with mobility issues*,” while another notes “*If I’ve got a problem, I don’t want to wait a month to be able to see my doctor*.” In addition, participants emphasize embedding clear disclaimers and actionable next-step guidance, such as recommending a neurologist follow-up or explaining probability scores to prevent undue alarm. Collectively, these insights underscore that while users place primary trust in physicians, they view PARK as a valuable adjunct for early triage and awareness, particularly in contexts where care is delayed, costly, or geographically limited. To mitigate concerns about false positives or negatives, we explicitly clarify that PARK is a screening tool rather than a diagnostic system, designed to complement, not replace, expert evaluation. Taken together, these findings reinforce PARK’s foundation in user-centered design and highlight straightforward pathways for enhancing trust, transparency, and responsible integration into clinical workflows.

PARK’s primary users are likely to be older adults, who represent the population most affected by PD^57^. Given that many may use the tool independently at home, variability in recording conditions, including background noise, lighting, and camera positioning, is inevitable. Despite these challenges, PARK maintains stable performance across a range of video quality levels (Fig. 9b), underscoring its robustness to the kinds of imperfections commonly encountered in real-world environments. This resilience is critical for ensuring reliable assessments in unsupervised, at-home settings.

Manual review of false-positive cases reveals that failure to follow task instructions is a common contributing factor, occurring in 58% of such cases, most frequently during the finger-tapping task (47%). In several cases, annotators also observe Parkinsonian symptoms in individuals who did not report a PD diagnosis. Given that the data are collected via a publicly accessible web platform^58^, some users may test the system by mimicking PD-like symptoms, inadvertently confusing the model. Alternatively, some participants may have had undiagnosed PD, leading to inaccurate self-reporting. Although self-reported PD status can be reliable in remote cohorts^59,60^, reliance on self-reporting remains a limitation, particularly given that a substantial portion of individuals (*n* = 1102, 59.1%) self-report their diagnosis (among 670 individuals with PD, 290 (43.3%) are self-reported) in our dataset. To quantify the potential impact, we stratify the combined evaluation set into self-reported (*n* = 63) and clinically validated (*n* = 257) subgroups. Model accuracy is 77.8% [66.1, 86.3] for the self-reported group versus 80.9% [75.7, 85.3] for the clinically validated group (difference −3.2%, two-proportion *z* = − 0.565, *p* = 0.57). AUROC values are 0.83 [0.79, 0.86] and 0.86 [0.85, 0.86], respectively (difference −0.03, *p* = 0.09). Neither difference is statistically significant at the *α* = 0.05 level, and the wider confidence intervals for the self-reported group reflect its smaller sample size. Together, these results indicate that our model performs comparably across self-reported and clinically diagnosed cohorts, despite the inherent limitations of self-reporting.

Among the false-negative cases (*n* = 25), many individuals do not exhibit PD symptoms observable in the videos. Specifically, in 24% of these cases, no PD symptoms are visible across any of the task videos. Although we carefully selected multiple tasks covering speech, facial expression, and motor assessments, video-only analysis may not be comprehensive. Note that we do not benchmark PARK against gold-standard PD biomarkers such as DAT-SPECT or *α*-synuclein seeding assays, as we do not expect PARK to outperform these clinical biomarkers. Instead, PARK could enhance access to care for individuals, especially those from underserved communities, who would otherwise not seek PD care, as these assessments can be costly and unavailable to them. However, in future studies aiming to improve upon these clinical benchmarks, UFNet can be further trained with additional modalities, such as MRI brain scans, DAT-SPECT, or *α*-synuclein seeding assays.

Our study has several limitations. To begin with, data are collected across eight independent studies, each governed by distinct IRB protocols and timelines, resulting in procedural inconsistencies. Notably, three different diagnostic criteria are used to confirm PD, including the Movement Disorder Society (MDS) Clinical Diagnostic Criteria, the UK Parkinson’s Disease Society Brain Bank Criteria, and the Gelb criteria. These frameworks share core requirements (the presence of Parkinsonian motor symptoms and the exclusion of features suggestive of alternative diagnoses), but differ in their comprehensiveness and emphasis. While the use of different diagnostic criteria across the independent datasets is a potential source of heterogeneity, the use of formal diagnostic criteria nonetheless ensures higher diagnostic confidence than non-use or reliance on self-report. Next, UFNet requires all three task videos and cannot handle missing data. Although individuals with partial data (*n* = 950, 50.9%) are included in training task-specific models, they could not contribute to advancing the fusion model. The model also does not account for participants’ medication status (e.g., ON vs. OFF periods), which can affect symptom visibility. Future work may extend UFNet to support incomplete inputs and incorporate additional inputs, such as medication status, when available. In addition, although this study assembles the largest multi-task video dataset for PD screening to date (1865 unique participants), the quality of the collected data is a concern. In the near future, we plan to integrate automated quality and compliance checks to ensure the model is trained and evaluated on high-quality data. When poor-quality data is identified for any of the three tasks, the user can be prompted to re-record with actionable feedback. Another limitation is the dominance of participants from the United States. Although participants are recruited globally from 36 countries, the majority (*n* = 1751, 93.9%) are based in the U.S., which may limit the model’s fairness and global applicability. Despite the diversity of our dataset, many subgroups have small sample sizes, thereby limiting the power of the corresponding statistical tests. Therefore, some findings may reflect insufficient statistical power rather than a true effect. Finally, while PARK shows strong overall performance, there are noticeable performance declines in the most challenging real-world deployment scenario. Specifically, sensitivity and positive predictive value (PPV) are significantly lower in the external evaluation (unsupervised) cohort compared to the other two settings. We hope that additional quality and compliance checks will improve data quality for unsupervised recordings, thereby enhancing PARK’s reliability when used independently at home.

In summary, this study introduces a clinically promising, user-aligned framework for AI-driven remote screening for PD. By validating PARK across diverse cohorts, deployment conditions, and usability contexts, we demonstrate that scalable, multimodal tools can achieve promising accuracy while remaining accessible to underserved populations. Beyond technical performance, real-world adoption of digital health technologies depends on usability, equity, and safety. PARK addresses these dimensions through robust design choices, including task-specific uncertainty modeling and user-informed evaluation. As healthcare systems increasingly shift toward remote and preventive care, tools such as PARK offer a viable path forward by lowering barriers to initial neurological assessment and informing the development of inclusive digital health solutions.

## Supplementary information

Transparent Peer Review file

Supplementary Information

## Data Availability

The recorded videos are collected using a web-based tool. The tool is publicly accessible at https://parktest.net^26^. The datasets used in this study consist of participant videos and associated clinical information collected under institutional review board (IRB) approval across multiple studies. Because these data contain sensitive health information and identifiable video recordings, they cannot be made publicly available due to IRB restrictions and compliance with the Health Insurance Portability and Accountability Act (HIPAA). Access to the data may be considered for collaborative research purposes and will be subject to approval under the relevant IRB protocols and a data use agreement. Researchers interested in accessing the data for collaboration may contact the principal investigator, Ehsan Hoque (mehoque@cs.rochester.edu), to discuss potential data-sharing arrangements that are consistent with ethical and regulatory guidelines. Such correspondence will be evaluated on a case-by-case basis, and we will aim to respond within 5 working days. All source data underlying the figures and tables in this study are publicly available through a repository on Figshare at https://doi.org/10.60593/ur.d.29410703^61^. The repository includes the numerical data used to generate the plots and summary statistics reported in the manuscript, along with documentation describing each file and its associated data columns. The UFNet dataset and preprocessing code used in this study are available at the GitHub repository https://github.com/ROC-HCI/UFNet (https://doi.org/10.5281/zenodo.18940836)^62^.

## Code availability

All custom code used in this study, including scripts for video processing, feature extraction, model training and evaluation, statistical analyses, and figure generation, is publicly available via the GitHub repository: https://github.com/saiful1105020/park_communications_medicine(https://doi.org/10.5281/zenodo.18940702)^63^. All data processing and analyses were performed using Python. The repository includes instructions for setting up the Python environment and the exact package versions required to reproduce the experiments and analyses reported in this study.
