# Supplementary material for: Validation of remote multimodal AI screening for Parkinson disease across diverse settings
Source: Commun Med (Lond). 2026 May 6;6:488. doi: 10.1038/s43856-026-01606-6 (PMC13586151; doi:10.1038/s43856-026-01606-6)
Supplement: Supplementary file 1 [file 43856_2026_1606_MOESM1_ESM.pdf]

nature portfolio

Peer Review File

Validation of Remote Multimodal AI Screening for Parkinson Disease Across Diverse Settings

Corresponding Author: Mr Md Saiful Islam

This file contains all reviewer reports in order by version, followed by all author rebuttals in order by version.

Version 0:

Reviewer comments:

Reviewer #3

(Remarks to the Author)
DEAR AUTHORS,
This study evaluates a large, multi-cohort population using remote video-based neurological tasks collected through an online platform. Participants completed three short webcam tasks including finger tapping, smile mimicry, and speech, which were analyzed using task-specific neural networks and combined through an uncertainty-aware fusion model. Across internal and external evaluation cohorts, including supervised and unsupervised home settings, the reported sensitivity and specificity fall in a moderate range and are generally consistent with prior work on remote Parkinson's disease screening rather than exceeding current state of the art.

The authors have appropriately addressed the reviewers' concerns through additional analyses, including uncertainty-stratified performance and an expanded post hoc interpretability analysis, as well as a clearer discussion of limitations. While the explainability remains at a high level, it provides some insight into the relative contribution of tasks and features without overclaiming clinical interpretability. The manuscript is balanced, clearly written, and appropriately frames the system as a screening rather than diagnostic tool. Overall, the study is methodologically sound and likely to be of interest to the digital neurology and translational medicine community, and I recommend it for publication.

Open Access This Peer Review File is licensed under a Creative Commons Attribution 4.0 International License, which permits use, sharing, adaptation, distribution and reproduction in any medium or format, as long as you give appropriate credit to the original author(s) and the source, provide a link to the Creative Commons license, and indicate if changes were made.
In cases where reviewers are anonymous, credit should be given to 'Anonymous Referee' and the source.
The images or other third party material in this Peer Review File are included in the article's Creative Commons license, unless indicated otherwise in a credit line to the material. If material is not included in the article's Creative Commons license and your intended use is not permitted by statutory regulation or exceeds the permitted use, you will need to obtain permission directly from the copyright holder.
To view a copy of this license, visit https://creativecommons.org/licenses/by/4.0/
