# Supplementary material for: Validation of remote multimodal AI screening for Parkinson disease across diverse settings
Source: Commun Med (Lond). 2026 May 6;6:488. doi: 10.1038/s43856-026-01606-6 (PMC13586151; doi:10.1038/s43856-026-01606-6)
Supplement: Supplementary file 2 [file 43856_2026_1606_MOESM2_ESM.pdf]

Supplementary Information: Validation of Remote Multimodal AI Screening for Parkinson Disease Across Diverse Settings

Md Saiful Islam[1,2*†], Tariq Adnan[1,2†], Abdelrahman Abdelkader[1†], Zipei Liu[1], Evelyn Ma[1], Sooyong Park[1], Asif Azad[2,7], Pai Liu[1], Meghan Pawlik[3], Emily Hartman[3], Erin Shelton[4], Kristina B. Larson[5], M Saifur Rahman[2], Cathe Schwartz[4], Karen Jaffe[4], Jamie L. Adams[3], Ruth B. Schneider[3], Jan Freyberg[6], E. Ray Dorsey[3,7], Ehsan Hoque[1,8]

[1]University of Rochester, Rochester, United States.
[2]Bangladesh University of Engineering & Technology, Dhaka, Bangladesh.
[3]University of Rochester Medical Center, Rochester, United States.
[4]InMotion, Beachwood, United States.
[5]Harvard Medical School, Boston, United States.
[6]Google Research, London, United Kingdom.
[7]Atria Health and Research Institute, New York, United States.
[8]Ministry of Defense, Riyadh, Saudi Arabia.

*Corresponding author(s). E-mail(s): mislam6@ur.rochester.edu;
Contributing authors: tadnan@ur.rochester.edu; aabdelka@u.rochester.edu; Jamie_Adams@urmc.rochester.edu; Ruth_Schneider@urmc.rochester.edu; janfreyberg@google.com; Ray.Dorsey@chet.rochester.edu; mehoque@cs.rochester.edu;
†These authors contributed equally

1 Survey Questions for User-centered Evaluation

**Survey Questions Administered to Participants (Response formats indicated per section)**
**Usability** (1-5 points scale)
Strongly Disagree (1) / Disagree (2) / Neutral (3) / Agree (4) / Strongly Agree (5)
1. The instructional videos helped me to complete the tasks.
2. The tasks were easy to complete.
3. It was easy to interact with PARK-Bot.
4a. I think that I would like to use the PARK tool frequently.
4b. I found the PARK tool unnecessarily complex.
4c. I thought the PARK tool was easy to use.
4d. I think that I would need the support of a technical person to be able to use the PARK tool.
4e. I found the various functions in the PARK tool were well integrated.
4f. I thought there was too much inconsistency in the PARK tool.
4g. I would imagine that most people would learn to use the PARK tool very quickly.
4h. I found the PARK tool very cumbersome to use.
4i. I felt very confident using the PARK tool.
4j. I needed to learn a lot of things before I could get going with the PARK tool.
**Utility and Comprehensiveness of Resources** (1-5 points scale)
Strongly Disagree (1) / Disagree (2) / Neutral (3) / Agree (4) / Strongly Agree (5)
5. The description of the risk of demonstrating features of Parkinson's disease was easy to understand.
6. The description of the risk of demonstrating features of Parkinson's disease was informative.
7. The severity ratings provided for the speech, facial expression, and motor tasks were easy to understand.
8. The severity ratings provided for the speech, facial expression, and motor tasks were informative.
9. PARK Chatbot adequately answered my questions.
10. The resources provided were informative.
11. The resources provided were comprehensive.
**Preference Compared to Clinical Appointments** (1-5 points scale)
Less Preferred (1) / Somewhat Less Preferred (2) / About the Same (3) / Somewhat More Preferred (4) / More Preferred (5)
12. In terms of accessibility, how does using the PARK tool compare to having an appointment at a medical facility?
13. In terms of convenience, how does using the PARK tool compare to having an appointment at a medical facility?
14. How would you rate your level of comfort when using the online assessment tool compared to being assessed in a medical facility?
15. How would you rate your level of privacy when using the online assessment tool compared to being assessed in a medical facility?
16. I would prefer the overall financial impact of using an online tool compared to visiting a medical facility.
**Perceived Risks from Using the PARK Tool** (binary scale)
No (0) / Yes (1)
17. Viewing the results from PARK caused me to feel anxious.
18. Do you think the PARK tool responses could be harmful?
19. Do you think the PARK Chatbot responses could be harmful?
**Perceived Benefits from the PARK Tool** (1-5 points scale)
Strongly Disagree (1) / Disagree (2) / Neutral (3) / Agree (4) / Strongly Agree (5)
20. Viewing the results from PARK caused me to feel empowered.
21. Viewing the results from PARK caused me to feel in control of my health.
**Preference for using PARK Chatbot** (1-3 points scale)
Never (1) / Sometimes (2) / Often (3)
22. In terms of comfort, when would you prefer to speak to PARK Chatbot?
23. In terms of comprehensiveness, when would you prefer to speak to PARK Chatbot?
24. In terms of ease-of-use, when would you prefer to speak to PARK Chatbot?
25. In terms of safety, when would you prefer to speak to PARK Chatbot?
26. In general, when would you prefer to speak to PARK Chatbot?

Supplementary Table 1: **Survey questions used to evaluate usability, risk, benefit, and user preferences regarding the PARK tool.** The questions are grouped into six broad groups – usability, utility, preference for using the PARK tool, risks, benefits, and preference for using the Chatbot.

2 Hyper-parameter Search

**Hyperparameter**	**Values/range**	**Distribution**
batch size	$\{256, 512, 1024\}$	Categorical
learning rate	$[5e^{-5}, 1.0]$	Uniform
use minority oversampling?	{"yes", "no"}	Categorical
oversampling method	{"SMOTE", "SVMSMOTE", "ADASYN", "BoarderlineSMOTE", "SMOTEN", "RandomOversampler"}	Categorical
number of hidden layers	$\{1\}$	Categorical
projection dimension	$\{128, 256, 512\}$	Categorical
query (query/key/value) dimension	$\{32, 64, 128, 256\}$	Categorical
hidden dimension	$\{4, 8, 16, 32, 64, 128\}$	Categorical
dropout probability	$[0.05, 0.50]$	Uniform
η (UFNet's internal parameter)	$[0.1, 100]$	Uniform
number MC dropout rounds	$\{30\}$	Categorical
number of epochs	$[1, 300]$	Uniform Integer
optimizer	{"SGD", "AdamW", "RMSprop"}	Categorical
momentum	$[0.1, 1.0]$	Uniform
use scheduler?	{"yes", "no"}	Categorical
scheduler	{"step", "reduce on plateau"}	Categorical
step size	$[1, 30]$	Uniform Integer
patience	$[1, 20]$	Uniform Integer
gamma	$[0.5, 0.95]$	Uniform

Supplementary Table 2: **Hyper-parameter search space for the UFNet models.** We used the Weights & Biases tool for automated hyper-parameter tuning within this search space. Bayesian search was used instead of grid search for compute efficiency.

3 Criteria for Assessing Video Quality

**Quality Assessment Criteria**
**Finger-tapping Task**
1. Blurry or dark video, but hand is clear
2. Hand is unclear due to blurriness and poor contrast
3. Multiple persons' hands are visible but only participant is tapping
4. Multiple persons' hands are visible and the person(s) in the background is tapping
5. Participant seated far from the camera – chest/waist visible
6. Participant seated too far from the camera – knees/legs visible
7. Wrist partially/fully goes out of frame for a few moments, but 5+ taps with consecutive wrist visibility
8. Wrist goes out of frame for over 2 seconds during the tapping task
9. Taps 3-4 times less/more than instructed
10. Taps 5+ times less/more than instructed
11. **Miscellaneous noncompliance:** video recorded from phone, bad angle (not front-facing the camera), shaky camera, stayed idle entire time, did not attempt the task
**Smile Task**
1. Idle for over five seconds before/after task.
2. Idle for over five seconds during task completion
3. Blurry or dark video, but face clear
4. Face unclear
5. Multiple persons' faces visible
6. Minor glare from eyeglasses, but eyes remain visible
7. Major glare blocks eye
8. Laughing or smile too fast
9. Some body movement, but face remains in the frame
10. Face goes out of frame due to body movement
11. Participant seated far from the camera – chest/waist visible
12. Participant seated too far from the camera – knees/legs visible
13. **Miscellaneous noncompliance:** video recorded from phone, talking during the task, bad angle (not front-facing the camera), face not in frame, shaky camera, stayed idle entire time, did not smile
**Speech Task**
1. Noise but speech clear
2. Speech hard to hear
3. Unnatural short pauses, trying to remember the phrase
4. Unnatural long pauses and irrelevant words/sentences
5. 3+ repeated words
6. Utterance said multiple times
7. Missed 3-5 consecutive words
8. Missed entire sentence
9. Multiple speakers

Supplementary Table 3: **Criteria used to assess video quality.** Either a blue item or combination of multiple items in the list meant a video is of poor quality. A single black item meant the quality label was ambiguous. Absence of all the items meant good quality video.
